# Transcriptomic Analysis of Quinoa Reveals a Group of Germin-Like Proteins Induced by *Trichoderma*

**DOI:** 10.3389/ffunb.2021.768648

**Published:** 2021-12-01

**Authors:** Oscar M. Rollano-Peñaloza, Patricia A. Mollinedo, Susanne Widell, Allan G. Rasmusson

**Affiliations:** ^1^Instituto de Investigaciones Quimicas, Universidad Mayor de San Andrés, La Paz, Bolivia; ^2^Department of Biology, Lund University, Lund, Sweden

**Keywords:** *Chenopodium quinoa*, transcriptomics, germin-like proteins, *Trichoderma*, plant-fungi interactions

## Abstract

Symbiotic strains of fungi in the genus *Trichoderma* affect growth and pathogen resistance of many plant species, but the interaction is not known in molecular detail. Here we describe the transcriptomic response of two cultivars of the crop *Chenopodium quinoa* to axenic co-cultivation with *Trichoderma harzianum* BOL-12 and *Trichoderma afroharzianum* T22. The response of *C. quinoa* roots to BOL-12 and T22 in the early phases of interaction was studied by RNA sequencing and RT-qPCR verification. Interaction with the two fungal strains induced partially overlapping gene expression responses. Comparing the two plant genotypes, a broad spectrum of putative quinoa defense genes were found activated in the cultivar Kurmi but not in the Real cultivar. In cultivar Kurmi, relatively small effects were observed for classical pathogen response pathways but instead a *C. quinoa*-specific clade of germin-like genes were activated. Germin-like genes were found to be more rapidly induced in cultivar Kurmi as compared to Real. The same germin-like genes were found to also be upregulated systemically in the leaves. No strong correlation was observed between any of the known hormone-mediated defense response pathways and any of the quinoa-*Trichoderma* interactions. The differences in responses are relevant for the capabilities of applying *Trichoderma* agents for crop protection of different cultivars of *C. quinoa*.

## Background

*Trichoderma* is a genus of ascomycete fungi widely studied for its versatile interactions with other organisms. *Trichoderma* can feed or parasitize on other fungi, bacteria, oomycetes and nematodes (Harman et al., 2004; Verma et al., 2007). Several species of *Trichoderma* are also symbionts with plants and can promote plant growth by several, yet so far only partially known mechanisms. Strains of several symbiotic species in the *Trichoderma harzianum* species complex (e.g., *Trichoderma afroharzianum* T22 (Chaverri et al., 2015), previously called *T. harzianum*) are used commercially because they can substantially improve yields of several species of crops (Harman et al., 1989; Monte, 2001; Hermosa et al., 2012). The strain T22 can improve the soil nutrient availability to plants (Altomare et al., 1999), and several species of *Trichoderma* have also been shown to enhance plant growth through volatile compound emission (Lee et al., 2016; Jalali et al., 2017) and stimulate plant systemic defense responses (Harman et al., 2004; Mukherjee et al., 2013). Nevertheless, plants do not always benefit from these interactions as described for some maize cultivar in field trials and lab experiments (Harman, 2006). Plant growth inhibition by *T. harzianum* has also been observed in quinoa seedlings after six but not two days of axenic co-culture (Rollano-Peñaloza et al., 2018).

Quinoa (*Chenopodium quinoa* Willd.) is an emerging crop of great interest due to its nutritional values (Vega-Gálvez et al., 2010) and its resistance to hostile environmental conditions, especially salinity and drought (Ruiz et al., 2014; Bazile et al., 2016). Quinoa seeds are gluten-free, contain all essential amino acids and its composition (vitamins, antioxidants, fatty acids and minerals) is highly suitable for human nutrition (Repo-Carrasco et al., 2003). Quinoa has a high genetic diversity, e.g., >4,000 accessions have been registered by the Food and Agriculture Organization (Zurita-Silva et al., 2014). The high genetic diversity of cultivars is the result of many years of selection by the indigenous people of the Andean Altiplano, where quinoa may have been domesticated 7,000 years ago by pre-Columbian cultures (Dillehay et al., 2007).

Quinoa agricultural yields can be boosted by *Trichoderma* application, as previously described (Ortuño et al., 2013). However, the outcome of plant-*Trichoderma* interactions is not always beneficial. Plant genotype-specific growth inhibition by commercially available *Trichoderma* strains have been reported for lentils (Prashar and Vandenberg, 2017), tomato (Tucci et al., 2011) sugarbeet (Schmidt et al., 2020) and maize (Harman, 2006). Thus, the incompatibility of particular plants with particular biocontrol strains can lead to undesired agricultural losses. Therefore, there is a need to understand the genotype-specific mechanisms that determine beneficial plant growth effects upon treatment with biocontrol agents like T22. Among the beneficial plant growth mechanisms triggered by biocontrol agents and mycorrhiza, the activation of plant defenses is one of the most studied topics. Plant defenses activated by mycorrhiza have been shown to include expression of Germin-like proteins (GLPs) (Fiorilli et al., 2018), and plant-fungi protein-protein interaction studies have shown that GLPs interact with *Trichoderma* cellulases (Saravanakumar et al., 2018). However, no studies about effects of *Trichoderma* on GLPs expression have been reported.

GLPs are plant proteins that have been especially well studied in grains like barley. These proteins are characterized by being associated with several enzymatic activities including oxalate oxidase (OXO), superoxide dismutase (SOD), ADP-glucose pyrophosphatase/ phosphodiesterase (AGPPase) and polyphenol oxidase (PPO) (Zimmermann et al., 2006; Dunwell et al., 2008). A potential function of germin-like proteins (GLPs) is found in its OXO and SOD activities, which may play a key role in production of hydrogen peroxide (H_2_O_2_) during plant defense (Ilyas et al., 2016). Because of a potential importance of GLP in protecting plant cells from superoxide toxicity produced under pathogen attacks, germin or GLP genes (i.e., *HvOXO1*) have been inserted into dicot plants like rapeseed or peanut to enhance their pathogen resistance (Livingstone et al., 2005; Dunwell et al., 2008; Liu et al., 2015).

In this work, we have studied the molecular response of two *C. quinoa* cultivars that have been shown to experience plant growth inhibition when treated over longer time with *T. harzianum* BOL-12 and *T. afroharzianum* T22 in axenic co-cultures. The response of quinoa to BOL-12 and T22 in the early phases of interaction was studied by transcriptomic analysis and RT-qPCR verification. Overall, we observed that upon interaction with the two fungal strains, a broad spectrum of putative quinoa defense genes were activated in Kurmi but not in the Real cultivar.

## Methods

### Biological Materials

Seeds of quinoa (*Chenopodium quinoa* Willd.) cultivars Maniqueña Real (*Real*) and Kurmi were kindly supplied by PROINPA (Quipaquipani, Bolivia). *Trichoderma afroharzianum*, Rifai, T22, anamorph ATCC 20847 (Chaverri et al., 2015) was purchased from the American Type Culture Collection (Manassas, VA, USA). *Trichoderma harzianum* BOL-12QD (BOL-12) was isolated and provided by the Instituto de Investigaciones Farmaco-bioquímicas (IIFB-UMSA, La Paz, Bolivia) (Rollano-Peñaloza et al., 2018). The study was performed under pertinent institutional, national and international guidelines and legislation.

### Fungal Growth

T22 and BOL-12 were maintained on potato dextrose agar (BD-Difco, Detroit, USA) at 25°C. To isolate conidiospore suspensions, one ml of sterile water was added to two-week-old *Trichoderma* cultures on potato dextrose agar and collected spores were filtered through a sterile piece of cotton wool. The spores were washed twice with sterile H_2_O (Milli-Q, Merck Millipore, Burlington, MA, USA) and pelleted at 3700g for 5 min at 4 °C in an Allegra X-12R centrifuge (Beckman, Brea, CA, USA). Spores were resuspended in sterile H_2_O and kept at 4°C until experiments.

Germination of T22 and BOL-12 spores for *C. quinoa* treatment was performed as described by Yedidia, Benhamou (Yedidia et al., 1999) using 15 ml tubes shaken at 200 rpm for 18 h. The germinated spore suspension was washed twice by centrifugation as described above and finally resuspended in sterile H_2_O. The final spore concentration was adjusted to be 1 germinated spore/μl and verified by colony forming unit (CFU) counts in potato dextrose agar Petri dishes.

### Disinfection of *C. quinoa* Seedlings and Germination

Seeds of *C. quinoa* were soaked in commercial bleach (NaClO; 27 g/kg) for 20 min., followed by six rinses in sterile H_2_O. Immediately thereafter, the seeds were placed on sterile water agar (8 g/L) in Petri dishes and incubated in darkness at 24°C for 14 h (Rollano-Peñaloza et al., 2018).

### Co-culture of Quinoa and *T. harzianum* in Petri Dishes

Five germinated axenic seedlings of each cultivar Kurmi and Real with similar root length were aligned in a straight line on each 12 x 12 cm square Petri dishes containing 0.1X Murashige and Skoog Basal Salts Mixture (MS; Duchefa, Haarlem, The Netherlands), supplemented with 8 g/L agar. The Petri dishes were tilted 45° during growth with the agar/air interface facing upwards and seedlings having the roots pointing toward the bottom part of the Petri dish. The seedlings were incubated at 24°C for 4 h before treatment with T22 or BOL-12.

*C. quinoa* seedlings were treated by adding 10 μl [1 CFU/μl] of either T22 or BOL-12 germinated spore suspension on the neck of the primary root. Ten μl of sterile H_2_O was added to each seedling in the mock control group. After treatment, the seedlings were incubated at 24°C in a 16 h light /8 h dark photoperiod. Co-cultivation was done under fluorescent lights (Polylux XLr 30W, GE, Budapest, Hungary) at 50 μmol m^−2^ s^−1^ for 12 and 36 h.

### Seedling Growth Analysis

Hypocotyl length was analyzed from images taken with a Digital Camera Canon EOS Rebel T3. Measurements from the photographs were done with the segmented line tool of *ImageJ* 1.49 (Abramoff et al., 2004).

### Sample Collection and RNA Extraction

For RNA extraction quinoa seedling were sampled 12 and 36 h after *Trichoderma* treatment. Each treatment enclosed five plates containing five seedlings. To reduce inter-plate variability, one root from each of the five plates was pooled for a biological replicate into pre-weighed aluminum foil envelopes. Roots were excised at the root-hypocotyl interface with a scalpel. The envelopes were weighed on a precision balance and shock-frozen in liquid nitrogen. Frozen samples were either processed immediately or stored at −80°C until RNA extraction. Roots and shoots were pooled separately.

Total RNA was extracted using the RNeasy Plant Mini Kit (Qiagen, Valencia, CA, USA), with the following modification: Root tissue samples preserved in liquid nitrogen were placed in a precooled mortar containing liquid nitrogen followed by thoroughly grinding without letting the samples thaw. Then 450 μL of Buffer RLT (Qiagen, Valencia, CA, USA) supplemented with B-mercaptoethanol (1%) was added. Grinding continued until samples thawed and were transferred to a 1.5 ml microcentrifuge tube. The rest of the procedure was followed according to Qiagen instructions. Total RNA quantity and quality was determined with a NanoDrop spectrophotometer. DNase treatment was performed with the DNA-*free* kit (Ambion, Carlsbad, CA, USA), following the instructions of the manufacturer. The integrity and quality of the RNA was determined as follows: 500 ng of DNase-treated RNA were dissolved in 8 μl of sterile water and split in two aliquots, one placed on ice and the other placed at 37°. After 20 min incubation, 2 μl of loading buffer was added to each sample and both aliquots were loaded on an agarose gel (2%) stained with ethidium bromide. The gel was run at 80 V for 30 min and visualized in an UV-transilluminator. Samples with sharp 18S and 28S rRNA and showing no evidence of degradation were retained.

### RNA-Seq Library Construction and Sequencing

Total RNA treated with DNase was sent to IGA technology services (IGA, Udine, Italy; http://www.igatechnology.com) for poly(A)^+^ mRNA purification, strand-specific cDNA synthesis, library construction (Truseq stranded mRNA-seq) and sequencing using a HiSeq2500 (Illumina Inc., San Diego, CA, USA) in paired-end mode with a read length of 125 bp. Raw sequences have been deposited at the National Center of Biotechnology Information (NCBI) under project accession number: PRJNA720675.

### Transcriptomic Analysis

RNA-seq reads were checked for quality by FastQC (v.0.9.0) and mapped on the quinoa genome “Kd” (Yasui et al., 2016a) and to the QQ74 coastal genome (Jarvis et al., 2017) by Tophat2 (v.2.2.9). Transcript abundances were assessed with HTSeq (v.0.9.1) with “intersection-nonempty” mode. Genes that had a minimum of 1 read mapped in each of the samples considered for analysis were included. Gene expression levels were measured as counts per million (CPM) (Anders et al., 2013). Library size normalization was performed using the trimmed mean of *M*-values (TMM) within the R package edgeR (v.3.14.0) (Robinson et al., 2010; Dillies et al., 2013). CPM were TMM-normalized in order to compensate for library size differences. Differential gene expression analysis comparing mock-treated samples with samples treated with *Trichoderma* was performed using edgeR with TMM normalized libraries (Anders et al., 2013) with a false discovery rate (FDR) of 5% (q < 0.05) (Benjamini and Hochberg, 1995).

### Functional Annotation of Differentially Expressed Genes

Gene ontology (GO) term enrichment for sets of differentially expressed genes were estimated with Argot2 through sequence function prediction (Falda et al., 2012). Singular enrichment analysis (SEA) for biological processes was performed with AgriGO v2.0 (Du et al., 2010). The statistical test for SEA was Fisher's exact test and for false discovery rate the Yekutieli method was applied (Tian et al., 2017).

### cDNA Synthesis and Gene Expression by qRT-PCR Analysis

Synthesis of cDNA was carried out with 500 ng of total RNA added to each 20 μl reaction of the RevertAid H Minus Reverse Transcription Kit (Thermo Scientific). The cDNA samples were stored at −20°C for downstream analysis. qRT-PCR of plant RNA was performed in a CFX384 Touch Real-Time PCR system (Bio-Rad, Hercules, CA, United States) using Maxima SYBR Green qPCR Master Mix (Thermo Scientific) supplemented with 0.25 μM of each specific primer and 10 ng of cDNA as template in a total volume of 10 μl/reaction. The PCR program had the following conditions: 1 cycle of: 95°C, 20 s; 30 cycles of: (95° C, 15 s; 60°C, 20s; 72 °C, 20s). The specificity of each PCR amplification was determined by melting curve analysis and by analysis in 2% agarose gels. The primer sequences can be found in Supplementary Table 7. The relative transcript expression was calculated by the Pfaffl algorithm, using *CqACT2A* and *CqMON1* as endogenous genes for normalization. *CqACT2A* and *CqMON1* were selected based on their stable expression as reference genes in *Arabidopsis (Wallström et al.*, *2012**)*. Ten-fold dilutions of cDNA template were used to determine the amplification efficiency for each gene (Pfaffl, 2001).

Primer pairs were designed using Perlprimer (Marshall, 2004) so that one of the primers in each pair spanned an exon-exon border, and the primer pairs were additionally checked using Netprimer (premierbiosoft.com) to avoid primer-primer interactions.

### Evolutionary Analysis of GLP

Protein alignments were made using Muscle (Edgar, 2004). Aminoacid substitution models were evaluated by MEGA X (Kumar et al., 2018). The protein evolutionary tree was performed by maximum likelihood using LG model (Le and Gascuel, 2008) with gamma distribution (LG + G) and 95% limit for partial gaps. Total positions in the final dataset were 191. Bootstrap testing was conducted with 1000 replicates.

## Results

### Transcriptome Sequencing of *C. quinoa* in Axenic Co-culture With *Trichoderma*

Two cultivars of quinoa were grown axenically and subjected to 36 h treatments with the *Trichoderma* strains T22 and BOL-12 for gene expression analyses to detect molecular responses. During this short-term treatment, the effect of *Trichoderma* on seedlings was not measurable, consistent with previous observations (Rollano-Peñaloza et al., 2018).

Five RNA samples from quinoa roots treated with *Trichoderma* for 12 and 36 h were collected. The three RNA samples at 12 h post inoculation (hpi) showing best quantity and quality parameters were selected for RNA-seq. Similarly, three RNA samples at 36 hpi were selected to be analyzed with qRT-PCR. Sequencing was carried out in paired-end mode, and the final number of reads that passed the quality control varied between 10.2 and 23.1 million paired-end reads of 125 bp per sample (Table 1). Reads were mapped to the draft quinoa genome of cultivar Kd as well as to the chromosome-level assembly of the quinoa genome cultivar QQ74 (Table 1). On average, the proportion of mapped reads was substantially increased when reads were mapped to the QQ74 quinoa genome (93.9 %), as compared to the draft Kd quinoa genome (71.8%). Therefore, all downstream analyses were performed with data mapped to the QQ74 genome.

**Table 1:** RNA-seq summary of read numbers mapped to two quinoa reference genomes.

**Quinoa cv**.	**Treatment**	**Sample**	**Total reads^a^**	**Mapped reads^b^**	**%**	**Mapped reads^c^**	**%**	**Unique reads^d^**	**%**	**Multi-reads^e^**	**%**
Kurmi		1	12,609,683	8,902,151	70.6	11,717,393	92.9	10,428,786	89.0	1,288,607	11.0
	Mock	2	13,360,579	9,462,168	70.8	12,431,446	93.0	11,075,758	89.1	1,355,688	10.9
		3	14,186,924	10,144,341	71.5	13,301,928	93.8	11,987,944	90.1	1,313,984	9.9
Kurmi		1	13,017,241	9,321,854	71.6	12,231,647	94.0	1,095,2241	89.5	1,27,9406	10.5
	BOL-12	2	11,571,126	8,301,337	71.7	10,906,882	94.3	9,758,090	89.5	1,148,792	10.5
		3	14,321,132	10,211,966	71.3	13,449,878	93.9	12,018,768	89.4	1,431,110	10.6
Kurmi		1	12,371,536	8,500,983	68.7	11,225,637	90.7	9,860,686	87.8	1,364,951	12.2
	T22	2	11,390,953	8,151,138	71.6	10,691,577	93.9	9,582,419	89.6	1,109,158	10.4
		3	13,144,501	9,423,642	71.7	12,345,436	93.9	11,154,456	90.4	1,190,980	9.6
Real		1	14,414,857	10,423,990	72.3	13,597,954	94.3	12,178,545	89.6	1,419,409	10.4
	Mock	2	14,949,701	10,780,918	72.1	14,143,244	94.6	12,694,852	89.8	1,448,392	10.2
		3	13,625,079	9,759,644	71.6	12,839,479	94.2	11,778,409	91.7	1,061,070	8.3
Real		1	10,245,775	7,405,797	72.3	9,599,526	93.7	8,553,892	89.1	1,045,634	10.9
	BOL-12	2	11,350,821	8,261,409	72.8	10,425,272	91.8	9,259,149	88.8	1,166,123	11.2
		3	12,542,586	9,091,486	72.5	11,767,298	93.8	10,725,657	91.1	1,041,641	8.9
Real		1	23,140,059	17,009,224	73.5	21,733,959	93.9	19,385,551	89.2	2,348,408	10.8
	T22	2	14,568,308	10,646,741	73.1	13,743,816	94.3	12,645,961	92.0	1,097,855	8.0
		3	11,039,194	8,047,239	72.9	10,305,738	93.4	9,304,963	90.3	1,000,775	9.7

a *Total reads that passed the quality control per biological replicate in each treatment*.

b *Average between right and left reads mapped with Tophat2 to the inbred Kd quinoa genome (Yasui et al., 2016b)*.

c *Average between right and left reads mapped with Tophat2 to the QQ74 coastal quinoa genome (Jarvis et al., 2017)*.

d *Unique reads mapped to the QQ74 coastal quinoa genome*.

e *Reads mapped to multiple positions*.

### Differential Gene Expression in Quinoa in Response to *Trichoderma* Treatment

The differential gene expression analysis considered only reads that mapped to unique locations in the QQ74 genome. The average number of reads that were mapped to unique locations in the QQ74 genome was 89.8% (Table 1). The remaining reads (10.2%) producing multiple alignments were discarded. Further, only quinoa genes with at least one read in each of the samples analyzed were considered (Table 2).

**Table 2:** Differentially expressed genes in quinoa roots treated with *Trichoderma*.

**Quinoa**	**Experiment**	**Induced**	**Repressed**	**Total**	**Ind/Repr**	**Genes evaluated^a^**
Kurmi	BOL-12 vs. mock-treated	158	38	196	4.2	25 273
Kurmi	T22 vs. mock-treated	1,417	1,727	3,144	0.8	25 379
Real	BOL-12 vs. mock-treated	277	76	353	3.6	30 108
Real	T22 vs. mock-treated	1,170	139	1,309	8.4	30 745

a *Genes included had at least one read in each of the samples. The quinoa genome annotation contains 44,776 genes*.

Quinoa roots in general induced more genes than they repressed upon interaction with *Trichoderma*, with the exception of Kurmi interacting with T22 where more genes were repressed than induced (Table 2). Kurmi treated with T22 showed 16 times more differentially expressed genes than in the treatment with BOL-12. Similarly, quinoa cv. Real treated with T22, compared to the mock-controls, had 5.5 times more differentially expressed genes than Kurmi in the treatment with BOL-12 (Table 2).

**Figure 1:**
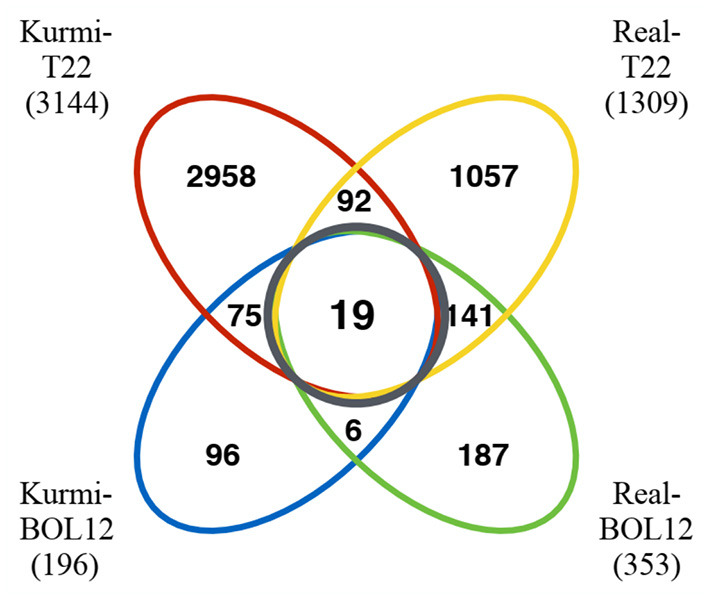
Venn diagram of quinoa genes differentially expressed in response to *Trichoderma*. Quinoa genes differentially expressed were grouped according to the cultivars and *Trichoderma* strains studied. The black circle indicates genes differentially expressed in both quinoa cultivars by each of the *Trichoderma* strains tested. The numbers in parenthesis indicate the number of genes differentially expressed in each of the quinoa-*Trichoderma* interactions studied.

Regarding communal effects by both *Trichoderma* strains, we observed more genes differentially expressed in cv. Real (141 genes) than in cv. Kurmi (75 genes) (Supplementary Tables 1–3). Among the quinoa genes up- or downregulated under one or several conditions, only 19 were communally differentially expressed in all experimental combinations, and all were induced (Figure 1, Table 3). That is, they were significantly induced during the interaction of each quinoa cultivar with each *Trichoderma* strain. The group of 19 differentially expressed genes were not significantly associated with any functional GO term upon analysis by SEA. However, the structural GO analysis suggests that 13 of the 19 gene products are localized outside the cytoplasm. This suggests an association of the response pattern to functions of the plasma membrane, the cell wall and the extracellular compartment, indicating functions relating to interactions with external stimuli (Table 3).

**Table 3:** Quinoa genes differentially expressed in both Kurmi and Real in response to both *Trichoderma* strains.

**Quinoa gene abbreviation**	**Quinoa gene code^a^**	**Gene name**	**Araport Code^b^**	**Quinoa protein length**	**Alignment length**	**Identity (%)**	**e-value**	**GO Cellular component**
*CqXTH6A*	AUR62024859	Xyloglucan endotransglucosylase/hydrolase 6	AT4G25810.1	285	284	70.1	3E-154	apoplast
*CqXTH6B*	AUR62024861	Xyloglucan endotransglucosylase/hydrolase 6	AT4G25810.1	286	284	71.5	1E-151	apoplast
*EXL4*	AUR62018945	Protein Exordium-like 4	AT4G08950.1	311	298	67.5	4E-143	cell wall
*CqPGIPA*	AUR62012077	Polygalacturonase inhibitor protein	AT5G06860.1	311	311	48.6	6E-82	cell wall
*CqPGIPB*	AUR62024339	Polygalacturonase inhibitor protein	AT3G12145.1	333	329	44.4	3E-80	cell wall
*CqChit1*	AUR62021382	Glycosyl hydrolase with chitinase insertion domain-containing protein	AT4G19810.2	364	368	47.0	3E-113	extracellular region
*CYP707A1*	AUR62010485	Abscisic acid 8'-hydroxylase 1	AT4G19230.1	450	465	74.2	0E+00	plasmodesmata
*PUB27*	AUR62013534	U-box domain-containing protein 27	AT5G64660.1	392	424	40.3	4E-87	plasmodesmata
*CqEP3.3*	AUR62031316	Basic endochitinase C. homolog of carrot EP3-3	AT3G54420.1	242	232	62.9	1E-104	plasma membrane
*NN*	AUR62029900	Protein of unknown function	AT1G68390.1	286	216	60.2	1E-96	plasma membrane
*AUR62005356*	AUR62005356	Transmembrane protein	AT1G23830.1	253	138	39.1	3E-22	plasma membrane
*SD25*	AUR62006585	G-type lectin S-receptor-like serine/threonine-protein kinase SD2-5	AT4G32300.1	831	789	35.1	4E-124	plasma membrane
*At1g35710*	AUR62039001	Probable leucine-rich repeat receptor-like protein kinase	AT1G35710.1	478	451	37.3	5E-66	plasma membrane
*CqHSP83a*	AUR62031424	Heat-shock protein 83	AT5G52640.1	579	443	92.6	0E+00	cytoplasm
*CqHSP83b*	AUR62021118	Heat-shock protein 83-like	AT5G52640.1	703	700	93.1	0E+00	cytoplasm
*CXE2*	AUR62014711	Probable carboxylesterase 2	AT1G47480.1	304	301	50.5	7E-109	cytosol
*AUR62011434*	AUR62011434	Protein of unknown function	AT2G26530.1	269	184	33.7	2E-16	intracellular
*CIGR1*	AUR62001765	Chitin-inducible GRAS family transcription factor	AT2G29060.2	690	626	43.3	1E-165	nucleus
*ERF071*	AUR62025525	Ethylene-responsive transcription factor ERF071	AT2G47520.1	249	217	42.9	3E-44	nucleus
CqWRKY33^*^	AUR62006298	WRKY33 transcription factor	AT2G38470.1	454	487	46.8	7E-116	nucleus

*All genes commonly differentially expressed in all plant-Trichoderma combinations were induced*.

a *Genes annotated in the Quinoa QQ74 genome (Jarvis et al., 2017) curated with information from their closest ortholog in A. thaliana*.

b *Gene codes from the Arabidopsis Information portal Araport*.

* *CqWRKY33 was included in the list of genes because significant difference respective to the control was confirmed by qRT-PCR (Figure 5)*.

### Quinoa Genes Differentially Expressed Unique to Each Cultivar

**Figure 2:**
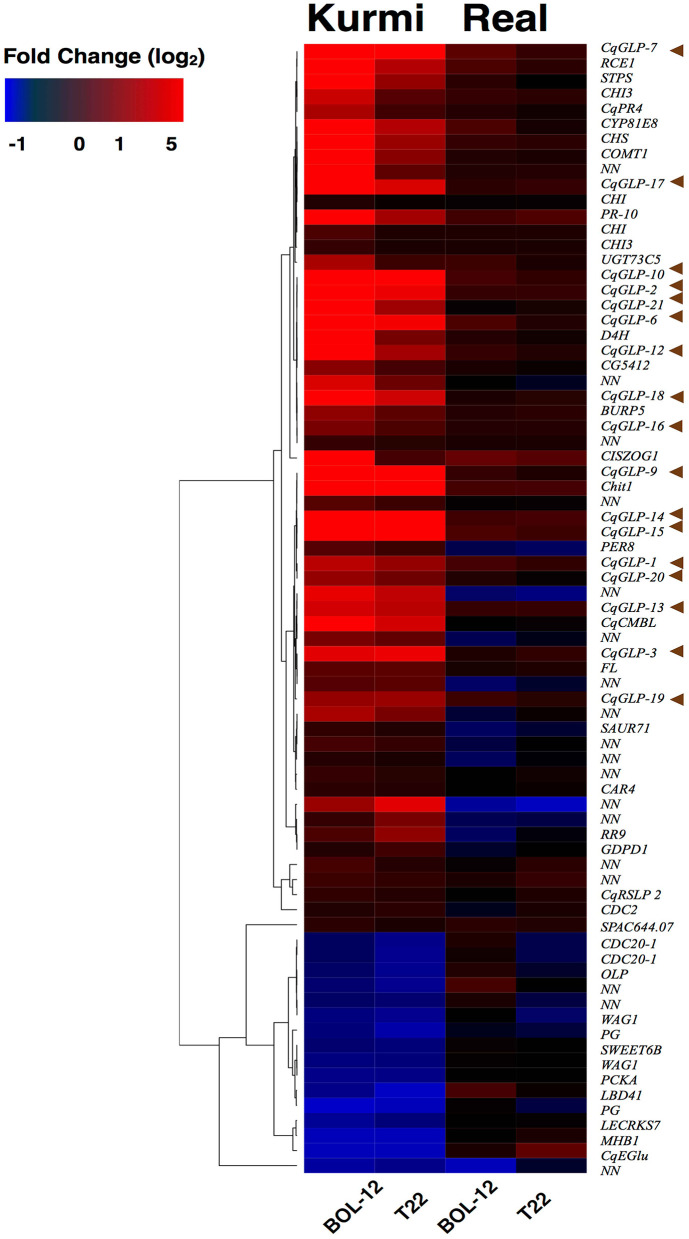
Heatmap profile of genes that respond to to *Trichoderma* BOL-12 and T22 in cv. Kurmi but not in *C. quinoa* cv. Real. Genes differentially expressed in cv. Kurmi in response to either *Trichoderma* strain, but not significantly responsive in cv. Real, were analyzed. Clustering by Euclidean distance shows the similarity in expressional change upon *Trichoderma* treatment. Brown arrows indicate *CqGLPs*.

We decided to analyze genes that were induced by *Trichoderma* and were uniquely expressed in each cultivar. The Kurmi cultivar upon interaction with either BOL-12 or T22, expressed 75 genes that were communally differentially expressed (DE) but were not differentially expressed upon either *Trichoderma* interaction in cv. Real (Figures 1, 2, Supplementary Table 1). The expression profiles of these genes were clustered by Euclidean distance and are shown in Figure 2. From the 75 DE genes in cv. Kurmi by both strains of *Trichoderma*, 59 genes were induced (Supplementary Table 1), whereas 16 DE genes were repressed (Supplementary Table 2). The 75 DE genes expressed in cv. Kurmi are expressed in cv. Real but are not responsive to the treatment with either of the *Trichoderma* strains (Figure 2).

Analysis of the 59 significantly induced genes revealed that 17 genes (*CqGLPs*) are highly expressed and share a high protein sequence identity (90%). These genes encode proteins that belong to the germin-like protein family (GLPs) (Figure 2; Supplementary Table 1). Further, several genes involved in flavonoid biosynthesis were specifically responsive in Kurmi. We identified 9 genes whose orthologs in *Arabidopsis thaliana* are described to be involved in the flavonoid biosynthesis pathway. These differentially expressed genes are orthologs to four out of five enzyme-coding genes necessary for production of flavonol glycosides from naringenin, also known as chalcone (Dao et al., 2011) (Figure 2, Supplementary Table 1).

The Real cultivar had 141 genes differentially expressed common to both *Trichoderma* strains tested (Figure 1, Supplementary Table 3). The cv. Real response to both *Trichoderma* strains showed mostly activation of transcription factors and enzymes without a significant match to a known pathway. Among the genes that were differentially expressed there are four ethylene-responsive transcription factors (ERFs), nine probable WRKY transcription factors and three chitinases (Supplementary Table 3).

Genes differentially expressed related to biotic interactions were observed in a larger proportion in quinoa cv. Kurmi than Real. Therefore, the focus of this study was on the response of the Kurmi cultivar.

### Functional Annotation of Differentially Expressed Genes

To assess the function of the differentially expressed genes we annotated the differentially expressed genes of all combinations with Gene Ontology (GO) terms for biological processes. The quinoa genome has 44 776 annotated genes (Jarvis et al., 2017) but the annotation with Argot only assigned GO terms to 50.5 % of the genes (i.e., 22 650 genes annotated with GO terms). Despite the low percentage of GO terms assigned, GO annotation for the biological process category in Kurmi plants treated with BOL-12 revealed defense response (GO:0006952) and response to biotic stimulus (GO:0009607) as the main and only processes associated to *Trichoderma* BOL-12 treatment (Supplementary Table 4). In contrast, the interaction between Kurmi and T22 did not show any significant GO term for biological processes.

Quinoa plants of the Real cultivar had more genes associated to GO terms than Kurmi. However, no specific association to a cluster of similar GO terms were observed (Supplementary Table 4). Specifically, Real treated with T22 showed 38 genes that were annotated as stress response genes (GO:0006950) and 6 genes that were annotated to chitin catabolic processes (GO:0006032) and associated redundant GO terms (Supplementary Table 4). Further, Real treated with BOL-12 did not show any GO terms directly associated to defense response or response to stress, yet highest significance was observed to GO terms for cell wall related processes (Supplementary Table 4). Nonetheless, in the interaction between Real and each strain of *Trichoderma* we observed several genes related to defense being commonly activated. Among them were WRKY transcription factors (nine differentially expressed genes), ethylene-responsive genes (4 differentially expressed genes) and chitinases (three differentially expressed genes) (Supplementary Table 3).

### Validation of RNA-Seq With qRT-PCR

**Figure 3:**
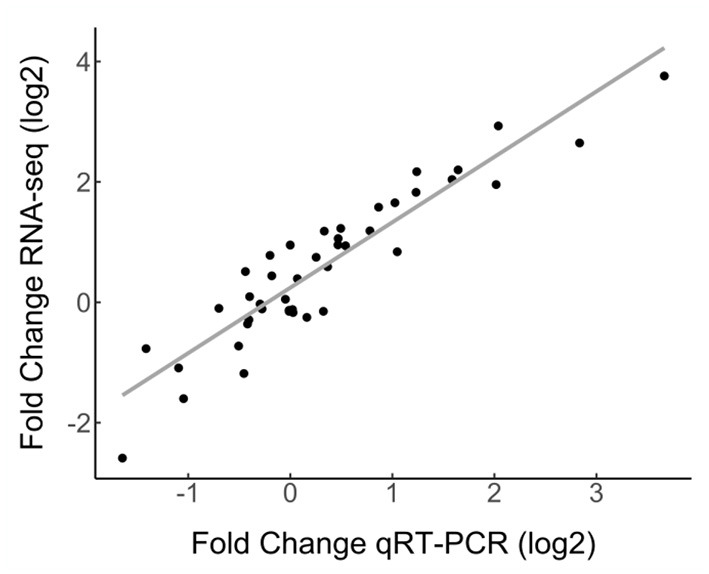
Correlation of RNA-seq and qRT-PCR gene expression data. Ten differentially expressed genes and two reference genes from the RNA-seq dataset were evaluated by qRT-PCR. Gene expression by qRT-PCR was normalized to the *CqAct2* reference gene. Fold change was measured by comparing samples treated with *Trichoderma* against mock- treated. The selected genes were assessed in all quinoa-*Trichoderma* combinations as averages of triple biological replicates. The Pearson correlation coefficient between the RNA-seq and qRT-PCR data was 0.921 (For data see Supplementary Table 5).

Quinoa root transcriptomes have not previously been analyzed. We therefore validated the gene expression data obtained by RNA-seq by performing qRT-PCR for 10 selected genes, including induced, repressed and stably expressed genes (Figure 3, Supplementary Table 5). A log-log linear model analysis of the RNA-seq data and the qRT-PCR data showed a strong correlation (R^2^) of 0.848. The correlation was higher when the different quinoa-*Trichoderma* interaction pairs were assessed independently (Supplementary Table 5).

### Changes in Root Gene Expression at 36 hpi

**Figure 4:**
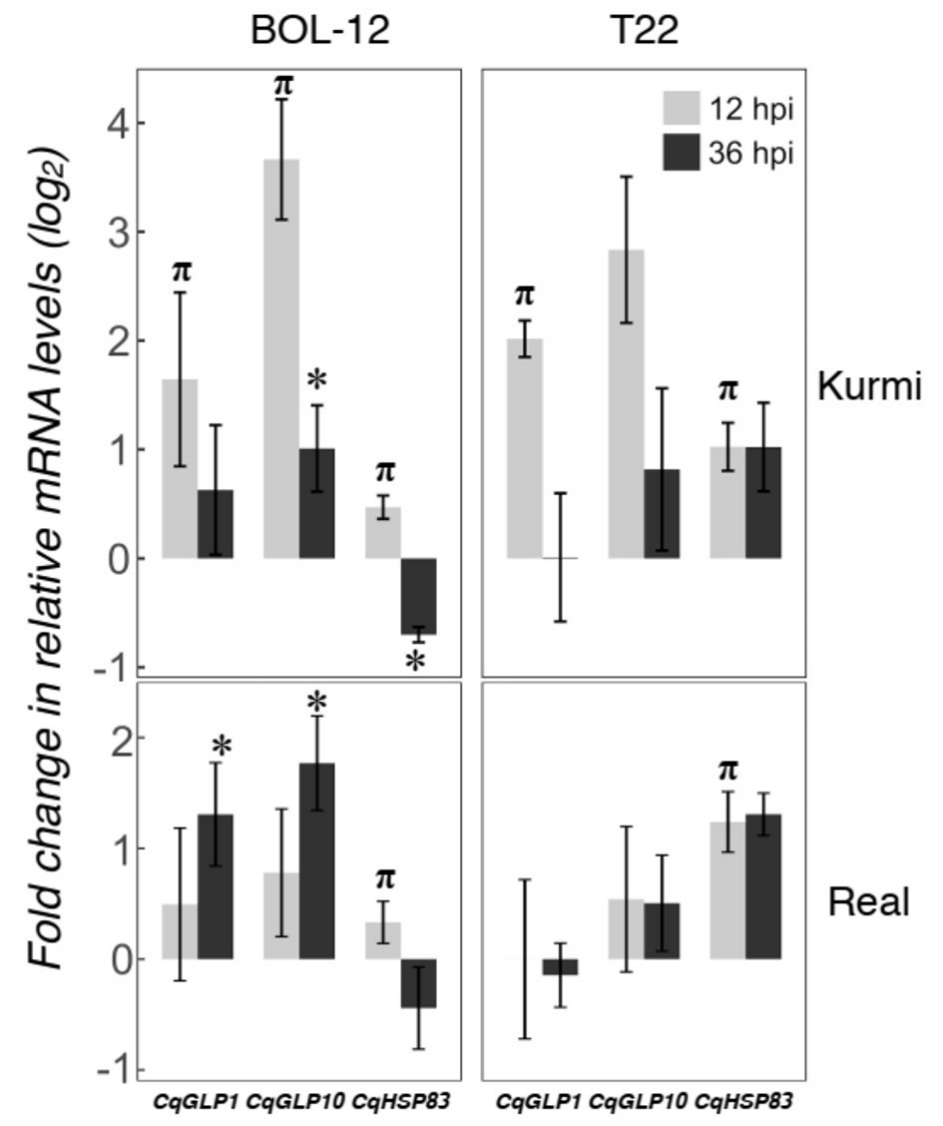
Gene expression at 12 and 36 hpi in representative *CqGLPs* and *CqHSP83* in quinoa roots treated with *Trichoderma*. Quinoa cv. Kurmi and cv. Real root samples treated with *Trichoderma* BOL-12 and T22 strains were assessed at 36 hpi by qRT-PCR. mRNA levels were normalized to the *CqAct2* reference gene. Fold changes (mean ± SE) were determined by comparing samples treated with *Trichoderma* against mock-treated ones. Asterisks denote significant changes in the gene expression as compared to the control treatment by qRT-PCR at 36 hpi (*p* < 0.05). Symbol π denotes significant changes in the gene expression as compared to the control treatment, using RNA-seq (*p* < 0.05) and confirmed by qRT-PCR (*p* < 0.05) at 12 hpi.

Time changes in the expression of quinoa genes by *Trichoderma* treatment were assessed by qRT-PCR at 36 hpi. We followed time-dependent changes in two highly induced genes (*CqGLP1* and *CqGLP10*) representing the GLP family and one gene that was induced in all quinoa-*Trichoderma* interactions (*CqHSP83)*. The gene expression of *CqGLP1* and *CqGLP10* was reduced at 36 hpi compared to 12 hpi in the Kurmi cultivar but its expression was still higher than the mock-treatment. In contrast, the gene expression of *CqGLP1* and *CqGLP10* in the Real cultivar was higher at 36 hpi than at 12 hpi, being statistically significant in the Real - BOL-12 interaction (Figure 4). The *CqHSP83* gene maintained its level of gene expression between 12 and 36 hpi by application of T22 in both cultivars. In contrast, the application of BOL-12 to both cultivars downregulated *CqHSP83* gene expression at 36 hpi as compared to 12 hpi. However, the downregulation was only significant in the Kurmi cultivar (Figure 4). Overall, the results suggest that the induction of the analyzed genes is slower in cv. Real than in cv. Kurmi.

### Shoot Gene Expression

**Figure 5:**
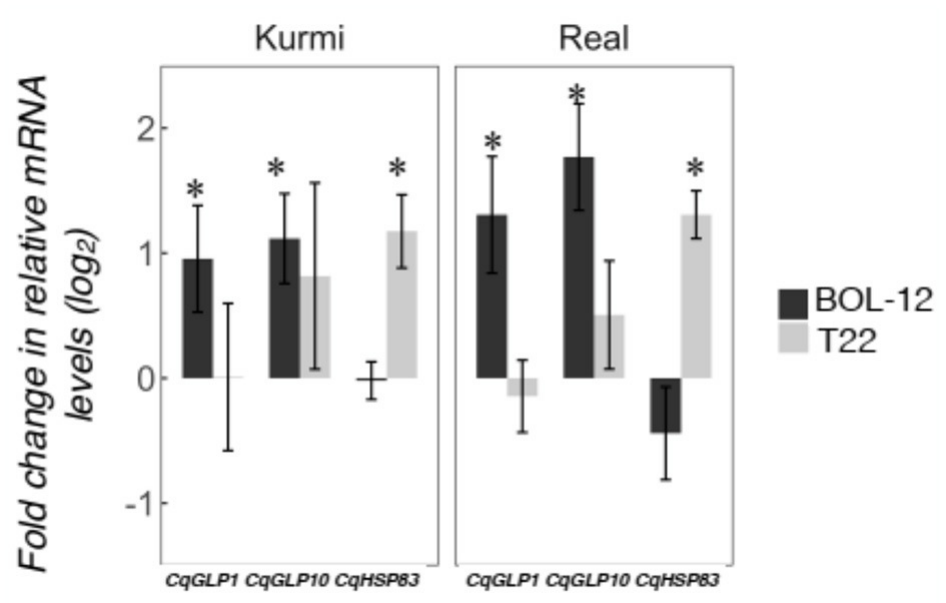
Gene expression changes in quinoa shoots after treatment of roots with *Trichoderma*. Quinoa shoot samples were assessed by qRT-PCR 36 h after treatment with *Trichoderma* to the roots. Gene expression was normalized to the *CqAct2* reference gene and is shown as mean ± SE. Asterisks denote significant changes in the gene expression as compared to the control treatment (*p* < 0.05). Two *CqGLP* genes that were induced in Kurmi roots at 12 hpi as well as the *CqHSP83* gene, which was induced in roots in all quinoa-*Trichoderma* interactions at 12 hpi are shown.

We investigated changes in the quinoa shoot gene expression 36 h after *Trichoderma* application to the root neck (Figure 5). Ten out of 12 genes investigated were also expressed in the shoots, *CqPER39* and *CqPR1C* gene expression was not detected in the shoots in any of the combinations studied (Supplementary Table 6). *Trichoderma*-induced gene expression changes in the shoots (Figure 5) showed a generally similar pattern of gene expression as observed in the roots at 36 hpi (Figure 4). *CqGLP1* and *CqGLP10* are significantly expressed in both cultivars upon interaction with BOL-12 but not with T22. Likewise, *CqHSP83* is significantly expressed in both cultivars when interacting with T22 but not when interacting with BOL-12 (Figure 5). The other genes did not show a significant correlation in the shoot-root expression in any of the quinoa-*Trichoderma* interactions studied (Supplementary Table 6).

### Evolutionary Analysis of the Germin-Like Proteins

**Figure 6:**
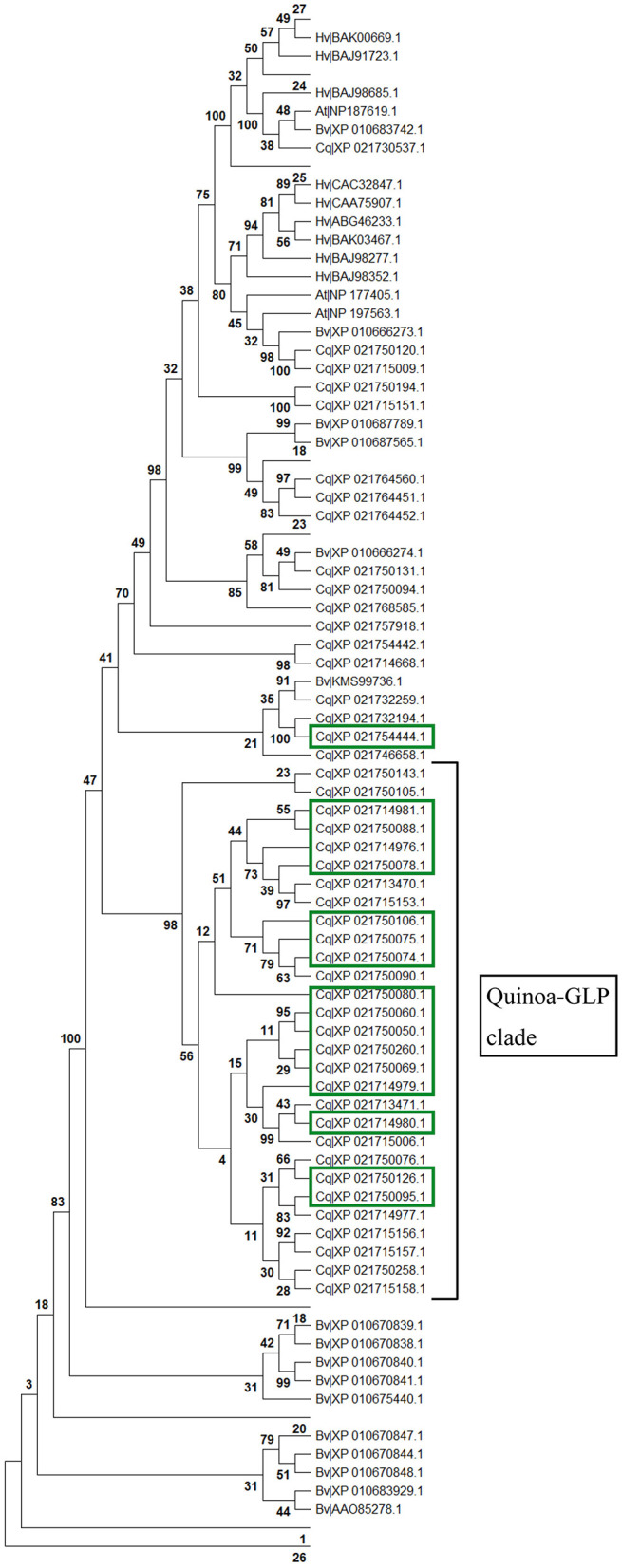
Protein evolutionary tree of germin and germin-like proteins. All identified germin-like proteins found in *C. quinoa* (Cq), *B. vulgaris* (Bv), *A. thaliana* (At) and *H. vulgare* (Hv) homologs were aligned by Muscle. The protein evolutionary tree was constructed by maximum likelihood using the LG + G model with 1,000 iterations. Bootstrap values are given in percentage (%). Values below 30% are not shown except for in the Trichoderma-responsive quinoa-specific GLP clade. The *Trichoderma*-induced homologs are marked in green.

Plant germins were first investigated and have been characterized in most functional detail in cereals (Ilyas et al., 2016). To investigate the coincidental induction of germin-like proteins in cv. Kurmi (Supplementary Table 1), we carried out BLAST searches to identify all germin and germin-like homologs in *C. quinoa, Beta vulgaris, A. thaliana* and *Hordeum vulgare* and performed alignments and evolutionary analyses. We found that 16 of the 17 quinoa GLPs induced by *Trichoderma* (highlighted in green) in cv. Kurmi belong to a single (98% bootstrap) *C. quinoa*-specific clade of 29 homologs (Figure 6, Supplementary Table 8). The remaining GLP induced by *Trichoderma* (*CqGLP20*) is grouped in an unresolved putative clade, which contains four non-expressed quinoa GLPs and one sugarbeet GLP (Figure 6). Quinoa germin-like proteins were significantly associated with specific homologs in *B. vulgaris*. Species-specific gene groups were also observed for *B. vulgaris* and *A. thaliana*. The result suggests that recent expansions of gene groups have occurred independently in the amaranth family species *C. quinoa* and *B. vulgaris*.

**Figure 7:**
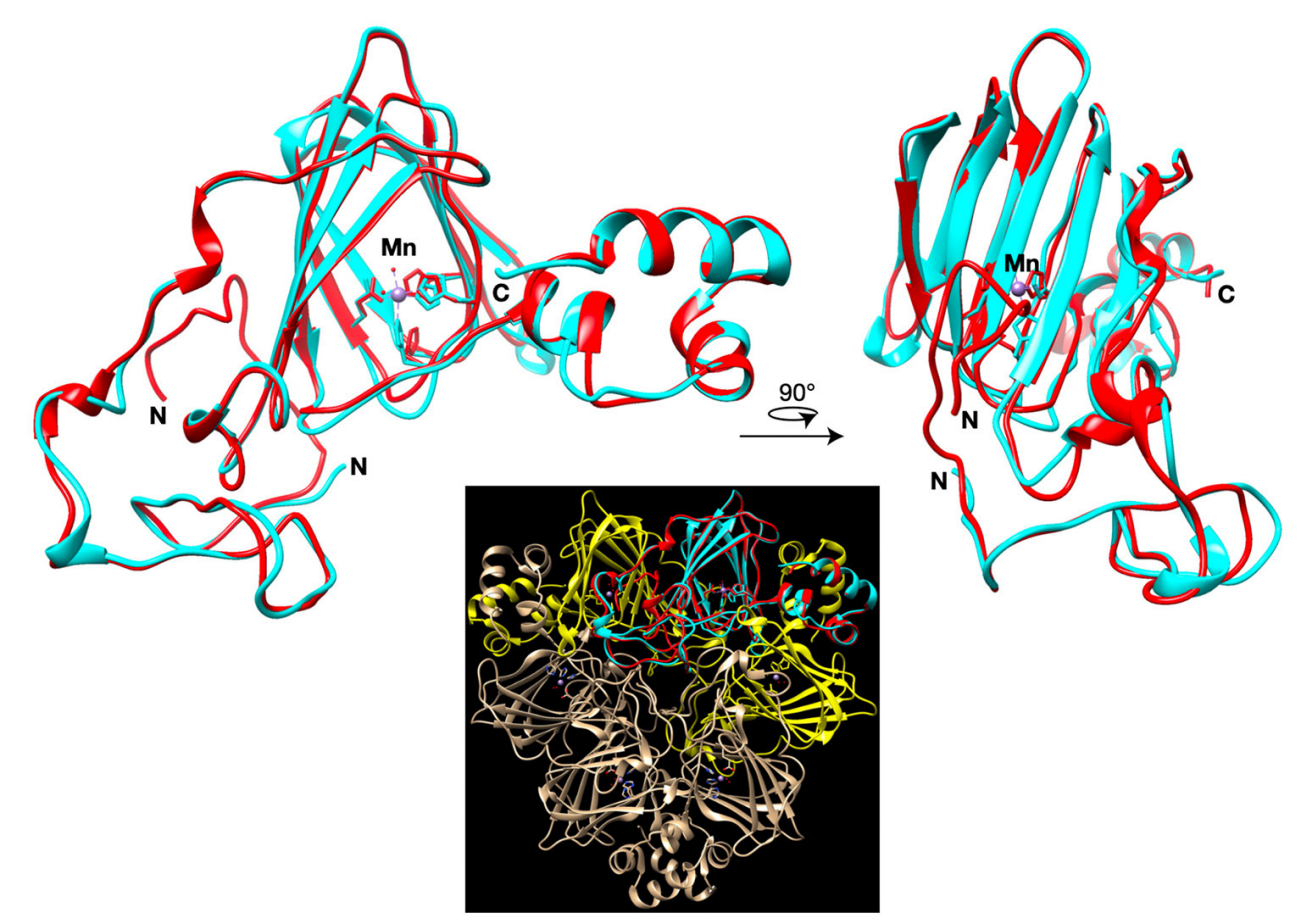
I-TASSER model of the *C. quinoa* GLP1. Whole quinoa germin-like protein (red) aligned to the template crystal structure of barley germin (oxalate oxidase; pdb:1FI2; cyan). The manganese ion of the active site is shown as a purple sphere. Inset shows the alignment integrated in the hexameric germin structure with neighboring germin monomers colored yellow.

Predicted structure of quinoa GLP1 shows a highly conserved structure compared to the crystal structure of Barley Germin (PDB: 1FI2), which has oxalate oxidase and manganese superoxide dismutase activities. The model suggest that the protein active site with aminoacid residues that bind to manganese is conserved and thus *CqGLP1* might have a potential enzymatic activity (Figure 7).

## Discussion

The outcome of plant-*Trichoderma* interactions with respect to both growth and physiological changes has been shown to be genotype-specific regarding both the plant and the *Trichoderma* biomaterial (Harman et al., 2004; Tucci et al., 2011). Here, we have observed a small set of quinoa genes being responsive in all combinations of *Trichoderma* strains and quinoa cultivars studied. However, we have found many more genes that are differentially expressed by a specific quinoa cultivar in response to either or both of two *Trichoderma* strains (Figure 1).

The set of 19 genes that showed significant responses in all cultivar-strain combinations mainly include genes connected to biotic stress response and cell wall modification (Table 3). Orthologs of these genes are known to be involved in defense response. For example, the polygalacturonase inhibitor protein *AtPGIP1* (AT5G06860) in *A. thaliana* is thought to inhibit cell wall pectin degrading enzymes, commonly produced by fungal pathogens (Lorenzo and Ferrari, 2002). Xyloglucan endotransglucosylase/hydrolases are cell wall repairing enzymes, many of which are induced by fungal infection (Miedes and Lorences, 2007). Further, two highly similar (protein sequence identity of 94.3%) heat-shock proteins (*CqHSP83A* and *CqHSP83B*) annotated to be involved in general stress responses (GO:0006950) were upregulated in all quinoa-*Trichoderma* interactions. Large heat-shock proteins (70–90 kDa) are known to be involved in plant defense response through the stabilization of protective plant proteins (Hubert et al., 2003; Takahashi et al., 2003; Zhang et al., 2015). The heat-shock proteins expressed by quinoa might have been induced to contribute to the stabilization of defense proteins that would prevent or counteract damages induced by *Trichoderma* (Rollano-Peñaloza et al., 2018).

Several differences in the defense response patterns between the quinoa cultivars were observed. Especially, the Kurmi cultivar displays specific activation of several homologs to biotic stress-associated plant genes (Supplementary Tables 1, 2). In contrast, the responsive genes in cv. Real were mostly involved in general cellular processes (Supplementary Table 3), and to a lesser extent involving defense response genes. The defense response gene set induced in cv. Real was also completely different from the one activated in Kurmi (Figure 1, Table 2, and Supplementary Tables 1–3). Surprisingly, in neither case an obvious association to known major pathogen response pathways like jasmonic acid, salicylic acid or ethylene pathways (Pieterse et al., 2009; Salas-Marina et al., 2011; Mathys et al., 2012) could be observed in the GO analysis at 12 hpi. The low association of the quinoa differentially expressed genes with these known pathways could, however, be caused by the relatively low level of GO annotation observed for the quinoa genome, which limits the statistical discovery power of the GO analysis for quinoa genes.

The quinoa genes specifically induced in the Kurmi cultivar upon interaction with *Trichoderma* (Supplementary Table 1) resemble a set of defense response genes observed in plant-pathogenic interactions (Dixon, 2001). Several of these induced genes (chalcone synthase, chalcone isomerase, flavonol synthase, UDP glycosyl transferase and *cis*-zeatin O-glucosyltransferase) belong to the flavonoid biosynthetic pathway (Dao et al., 2011). Flavonoids have an important role in plant defense (Dixon, 2001; Treutter, 2006). For example, some *A. thaliana* mutants lacking the UDP glycosyl transferase gene (*AtUGT74F1*) are more susceptible to *Pseudomonas syringae* infection than the wild-type (Boachon et al., 2014). Further, a QTL analysis for pathogen resistance in soybean identified two UDP glycosyl transferase genes as the candidate genes responsible for resistance to *Fusarium* (Cheng et al., 2017). Thus, the Kurmi cultivar might be producing flavonol glycosides in order to prevent damage from *Trichoderma* overgrowth.

The Kurmi cultivar specifically induced several plant defensins that belongs to the germin and GLP family (Figure 6 and Supplementary Table 1). The *Trichoderma*-responsive GLPs form a majority (16 genes out of 29) of a recently expanded quinoa-specific clade (Figure 6, Supplementary Table 8), which is thus strongly connected to *Trichoderma* interaction. Two of the GLP genes were further tested, and were found to be also induced in leaves (Figure 5). The timing of the induction further indicated that GLPs are induced in both Kurmi and Real, albeit more slowly in Real (Figure 4). Given that several quinoa GLPs were expressed upon interaction with *Trichoderma*, it is very likely that these GLP defensins have an important role in the plant immune response of quinoa. Resistance to microbe attacks has been previously connected to the speed of response in different cultivars (Pritsch et al., 2000; Venisse et al., 2002). Thus, the rapid induction of a cluster of GLPs in both roots and leaves of Kurmi compared to Real (Supplementary Tables 2, 3; Figure 4) makes these genes potential candidates for breeding to increase the tolerance to microbial attacks in quinoa plants.

The set of defense-related genes (peroxidases, chitinases, ERF and WRKY transcription factors) induced in quinoa cv. Real upon interaction with *Trichoderma* (Supplementary Table 3) has been observed in *L. japonicus* roots upon 1 h of incubation with chitin oligosaccharides (Nakagawa et al., 2011). However, the levels of such defense-related genes returned to normal after 7 h in *L. japonicus* whereas in quinoa remained induced after 12 h, possibly due to the persistance of interaction with the living *Trichoderma* agent as compared to the transient nature of the elicitor. Similar to the *L. japonicus system*, the 24 h interaction of *Trichoderma* with *A. thaliana* resulted in the induction of the same defense-related genes as seen here in quinoa (Brotman et al., 2013). This set of genes could thus be a basal gene response of plants after recognition of beneficial fungi like *Trichoderma* through chitin. In contrast, the Kurmi cultivar might have a different set of receptors that helps the plant to perceive a possible negative effect from *Trichoderma* and thus rapidly activate a different set of defense-related genes.

The plant root transcriptomic response to *Trichoderma* has been poorly studied compared to aerial parts (Vos et al., 2015). Nevertheless, it has been observed that in tomato roots the recognition of *Trichoderma* at 24 hpi activates ROS signaling, SA responses, cell wall modifications (Palma et al., 2019), JA responses and induction of plant defenses (Brotman et al., 2013; Ho et al., 2016). In our study, we have observed a similar pattern for ROS signaling, cell wall modifications and induction of plant defenses (Table 3, Supplementary Tables 1–3), confirming that the first response of root plants to beneficial fungi like *Trichoderma* is to activate defenses. Further, our study reveals that the defense response against beneficial fungi is variable between cultivars (Figure 2). The variable molecular response between cultivars to *Trichoderma* could help to create molecular markers of compatibility between certain plant cultivars and certain strains of *Trichoderma*.

In conclusion, our study suggests that *Trichoderma* triggers a defense response in quinoa plants. Comparing the defense response of two quinoa cultivars we can observe that the Kurmi cultivar mainly induced a set of genes involved in plant defense. In contrast, the Real cultivar did not have a clear response because most of the changes mediated by *Trichoderma* were related to general stress and regulation of biological processes. The Kurmi cultivar might have higher tolerance to microbe attacks due to the expression of genes involved in the biosynthesis of flavonol glycosides and a clade of GLP-defensins unique to quinoa. These genes are thus candidates for selection of quinoa cultivars with higher resistance to microbe attacks.

## Data Availability Statement

The datasets presented in this study can be found in online repositories. The names of the repository/repositories and accession number(s) can be found below: https://www.ncbi.nlm.nih.gov/bioproject/PRJNA720675.

## Author Contributions

AR and OR-P designed the study, analyzed the data, and wrote the paper with input from all authors who reviewed and approved the final manuscript. OR-P performed the experiments. All authors contributed to the article and approved the submitted version.

## Funding

This work was funded by the Swedish International Development Agency (SIDA) in a strategic collaboration between Universidad Mayor de San Andrés (UMSA) (Bolivia) and Lund University (Sweden), under the SIDA-UMSA Cooperation Agreement 75000554; the Lars Hiertas Minne Stiftelsen (OR) and the Jörgen Lindströms Stipendiefond (OR). Genome mapping, read count and blastn computations were performed on resources provided by SNIC (Swedish National Infrastructure for Computing) through Uppsala Multidisciplinary Center for Advanced Computational Science (UPPMAX, Uppsala, Sweden) under Project SNIC b2015011/b2016265 along with resources provided by UMSA (Universidad Mayor de Sán Andres) through Centro Nacional de Computación Avanzada para Bioinformática y Genómica (CnCaBo, La Paz, Bolivia).

## Conflict of Interest

The authors declare that the research was conducted in the absence of any commercial or financial relationships that could be construed as a potential conflict of interest.

## Publisher's Note

All claims expressed in this article are solely those of the authors and do not necessarily represent those of their affiliated organizations, or those of the publisher, the editors and the reviewers. Any product that may be evaluated in this article, or claim that may be made by its manufacturer, is not guaranteed or endorsed by the publisher.

## References

[B1] Abramoff M. D. Magalhães P. J. Ram S. J. (2004). Image processing with imageJ. Biophotonics Int. 11, 36–42.

[B2] Altomare C. Norvell W. A. Björkman T. Harman G. E. (1999). Solubilization of phosphates and micronutrients by the plant-growth-promoting and biocontrol fungus *Trichoderma harzianum* Rifai 1295-22. Appl. Environ. Microbiol. 65, 2926–33. 10.1128/AEM.65.7.2926-2933.1999 10388685 PMC91438

[B3] Anders S. McCarthy D. J. Chen Y. Okoniewski M. Smyth G. K. Huber W.. (2013). Count-based differential expression analysis of RNA sequencing data using R and Bioconductor. Nat Protoc. 8, 1765–86. 10.1038/nprot.2013.099 23975260

[B4] Bazile D. Jacobsen S. E. Verniau A. (2016). The Global expansion of quinoa: trends and limits. Front. Plant Sci. 7, 622. 10.3389/fpls.2016.00622 27242826 PMC4860459

[B5] Benjamini Y. Hochberg Y. (1995). Controlling the false discovery rate: a practical and powerful approach to multiple testing. J R Stat. Soc. Series B Stat. Methodol. 57, 289–300. 10.1111/j.2517-6161.1995.tb02031.x

[B6] Boachon B. Gamir J. Pastor V. Erb M. Dean J. V. Flors V. (2014). Role of two UDP-Glycosyltransferases from the L group of arabidopsis in resistance against *Pseudomonas syringae*. Eur. J. Plant Pathol. 139, 707–20. 10.1007/s10658-014-0424-7

[B7] Brotman Y. Landau U. Cuadros-Inostroza Á. Takayuki T. Fernie A. R. Chet I. (2013). *Trichoderma*-plant root colonization: escaping early plant defense responses and activation of the antioxidant machinery for saline stress tolerance. PLoS Pathog. 9, e1003221. 10.1371/journal.ppat.1003221 23516362 PMC3597500

[B8] Chaverri P. Branco-Rocha F. Jaklitsch W. Gazis R. Degenkolb T. Samuels G. J. (2015). Systematics of the *Trichoderma harzianum* species complex and the re-identification of commercial biocontrol strains. Mycologia. 107, 558–90. 10.3852/14-147 25661720 PMC4885665

[B9] Cheng P. Gedling C. R. Patil G. Vuong T. D. Shannon J. G. Dorrance A. E. (2017). Genetic mapping and haplotype analysis of a locus for quantitative resistance to *Fusarium graminearum* in soybean accession PI 567516C. Theor. Appl. Genet. 130, 999–1010. 10.1007/s00122-017-2866-8 28275816

[B10] Dao T. T. Linthorst H. J. Verpoorte R. (2011). Chalcone synthase and its functions in plant resistance. Phytochem Rev. 10, 397–412. 10.1007/s11101-011-9211-7 21909286 PMC3148432

[B11] Dillehay T. D. Rossen J. Andres T. C. Williams D. E. (2007). Preceramic adoption of peanut, squash, and cotton in northern Peru. Science. 316, 1890–3. 10.1126/science.1141395 17600214

[B12] Dillies M. A. Rau A. Aubert J. Hennequet-Antier C. Jeanmougin M. Servant N. (2013). A comprehensive evaluation of normalization methods for Illumina high-throughput RNA sequencing data analysis. Brief Bioinform. 14, 671–83. 10.1093/bib/bbs046 22988256

[B13] Dixon R. A. (2001). Natural products and plant disease resistance. Nature. 411, 843–7. 10.1038/35081178 11459067

[B14] Du Z. Zhou X. Ling Y. Zhang Z. Su Z. (2010). agriGO: a GO analysis toolkit for the agricultural community. Nucleic Acids Res. 38, W64–70. 10.1093/nar/gkq310 20435677 PMC2896167

[B15] Dunwell J. M. Gibbings J. G. Mahmood T. Saqlan Naqvi S. M. (2008). Germin and germin-like proteins: evolution, structure, and function. CRC Crit. Rev. Plant Sci. 27, 342–75. 10.1080/07352680802333938

[B16] Edgar R. C. (2004). MUSCLE: multiple sequence alignment with high accuracy and high throughput. Nucleic Acids Research. 32, 1792–7. 10.1093/nar/gkh340 15034147 PMC390337

[B17] Falda M. Toppo S. Pescarolo A. Lavezzo E. Camillo D. Facchinetti B.. (2012). A Argot2: a large scale function prediction tool relying on semantic similarity of weighted Gene Ontology terms. BMC Bioinformatics. 13, S14. 10.1186/1471-2105-13-S4-S14 22536960 PMC3314586

[B18] Fiorilli V. Vannini C. Ortolani F. Garcia-Seco D. Chiapello M. Novero M. (2018). Omics approaches revealed how arbuscular mycorrhizal symbiosis enhances yield and resistance to leaf pathogen in wheat. Sci. Rep. 8, 9625. 10.1038/s41598-018-27622-8 29941972 PMC6018116

[B19] Harman G. E. (2006). Overview of mechanisms and uses of *Trichoderma* spp. Phytopathology. 96, 190–4. 10.1094/PHYTO-96-0190 18943924

[B20] Harman G. E. Howell C. R. Viterbo A. Chet I. Lorito M. (2004). *Trichoderma* species — opportunistic, avirulent plant symbionts. Nat. Rev. Microbiol. 2, 43–56. 10.1038/nrmicro797 15035008

[B21] Harman G. E. Taylor A. G. Stasz T. E. (1989). Combining effective strains of *Trichoderma harzianum* and solid matrix priming to improve biological seed treatments. Plant Dis. 73, 631–7. 10.1094/PD-73-063130812563

[B22] Hermosa R. Viterbo A. Chet I. Monte E. (2012). Plant-beneficial effects of *Trichoderma* and of its genes. Microbiology. 158, 17–25. 10.1099/mic.0.052274-0 21998166

[B23] Ho C-. L. Tan Y-. C. Yeoh K-. A. Ghazali A-. K. Yee W-. Y. Hoh C-. C. D. (2016). novo transcriptome analyses of host-fungal interactions in oil palm (*Elaeis guineensis* Jacq.). BMC Genom. 17, 66. 10.1186/s12864-016-2368-0 26781612 PMC4717632

[B24] Hubert D. A. Tornero P. Belkhadir Y. Krishna P. Takahashi A. Shirasu K. (2003). Cytosolic HSP90 associates with and modulates the *Arabidopsis* RPM1 disease resistance protein. EMBO J. 22, 5679–89. 10.1093/emboj/cdg547 14592967 PMC275404

[B25] Ilyas M. Rasheed A. Mahmood T. (2016). Functional characterization of germin and germin-like protein genes in various plant species using transgenic approaches. Biotechnol. Letters. 38, 1405–21. 10.1007/s10529-016-2129-9 27230937

[B26] Jalali F. Zafari D. Salari H. (2017). Volatile organic compounds of some *Trichoderma* spp. increase growth and induce salt tolerance in Arabidopsis thaliana. Fungal Ecol. 29, 67–75. 10.1016/j.funeco.2017.06.007

[B27] Jarvis D. E. Ho Y. S. Lightfoot D. J. Schmöckel S. M. Borm L. i. B. (2017). The genome of *Chenopodium quinoa*. Nature. 542, 307–12. 10.1038/nature21370 28178233

[B28] Kumar S. Stecher G. Knyaz L. M. Tamura C. (2018). K. MEGA X: molecular evolutionary genetics analysis across computing platforms. Mol. Biol. Evol. 35, 1547–9. 10.1093/molbev/msy096 29722887 PMC5967553

[B29] Le S. Q. Gascuel O. (2008). An improved general amino acid replacement matrix. Mol. Biol. Evol. 25, 1307–20. 10.1093/molbev/msn067 18367465

[B30] Lee S. Yap M. Behringer G. Hung R. Bennett J. W. (2016). Volatile organic compounds emitted by *Trichoderma* species mediate plant growth. Fungal Biol. Biotechnol. 3, 7. 10.1186/s40694-016-0025-7 28955466 PMC5611631

[B31] Liu F. Wang M. Wen J. Yi B. Shen J. Ma J.. (2015). Overexpression of barley oxalate oxidase gene induces partial leaf resistance to Sclerotinia sclerotiorum in transgenic oilseed rape. Plant Pathol. 64, 1407–16. 10.1111/ppa.12374

[B32] Livingstone D. M. Hampton J. L. Phipps P. M. Grabau E. A. (2005). Enhancing resistance to *Sclerotinia minor* in peanut by expressing a barley oxalate oxidase gene. Plant Physiol. 137, 1354–62. 10.1104/pp.104.057232 15778458 PMC1088326

[B33] Lorenzo D. G. Ferrari S. (2002). Polygalacturonase-inhibiting proteins in defense against phytopathogenic fungi. Curr. Opin. Plant Biol. 5, 295–9. 10.1016/S1369-5266(02)00271-6 12179962

[B34] Marshall O. J. (2004). PerlPrimer: cross-platform, graphical primer design for standard, bisulphite and real-time PCR. Bioinformatics. 20, 2471–2. 10.1093/bioinformatics/bth254 15073005

[B35] Mathys J. Cremer D. e. Timmermans K. Van Kerkhove P. Lievens S. Vanhaecke B. M (2012). Genome-wide characterization of ISR induced in *Arabidopsis thaliana* by *Trichoderma hamatum* T382 against *Botrytis cinerea* infection. Front. Plant Sci. 3, 108. 10.3389/fpls.2012.00108 22661981 PMC3362084

[B36] Miedes E. Lorences E. P. (2007). The implication of xyloglucan endotransglucosylase/hydrolase (XTHs) in tomato fruit infection by *Penicillium expansum* Link. J. Agric. Food Chem. 55, 9021–6. 10.1021/jf0718244 17960871

[B37] Monte E. (2001). Understanding *Trichoderma*: between biotechnology and microbial ecology. Int. Microbiol. 4, 1–4. 10.1007/s101230100001 11770814

[B38] Mukherjee P. K. Horwitz B. A. Herrera-Estrella A. Schmoll M. Kenerley C. M. (2013). Trichoderma Research in the Genome Era. Annu. Rev. Phytopathol. 51, 105–29. 10.1146/annurev-phyto-082712-102353 23915132

[B39] Nakagawa T. Kaku H. Shimoda Y. Sugiyama A. Shimamura M. Takanashi K. (2011). From defense to symbiosis: limited alterations in the kinase domain of LysM receptor-like kinases are crucial for evolution of legume–*Rhizobium* symbiosis. Plant J. 65, 169–80. 10.1111/j.1365-313X.2010.04411.x 21223383

[B40] Ortuño N. Castillo J. Claros M. Navia O. Angulo M. Barja D. (2013). Enhancing the sustainability of quinoa production and soil resilience by using bioproducts made with native microorganisms. Agronomy. 3, 732–46. 10.3390/agronomy3040732

[B41] Palma D. e. Salzano M. Villano M. Aversano C. Lorito R. Ruocco M. M. (2019). Transcriptome reprogramming, epigenetic modifications and alternative splicing orchestrate the tomato root response to the beneficial fungus Trichoderma harzianum. Hortic. Res. 6, 5. 10.1038/s41438-018-0079-1 30603091 PMC6312540

[B42] Pfaffl M. W. A. (2001). New mathematical model for relative quantification in real-time RT-PCR. Nucleic Acids Res. 29, e45. 10.1093/nar/29.9.e45 11328886 PMC55695

[B43] Pieterse C. M. J. Leon-Reyes A. Van der Ent S. Van Wees S. C. M. (2009). Networking by small-molecule hormones in plant immunity. Nat. Chem. Biol. 5, 308–16. 10.1038/nchembio.164 19377457

[B44] Prashar P. Vandenberg A. (2017). Genotype-specific responses to the effects of commercial *Trichoderma* formulations in lentil (*Lens culinaris* ssp. *culinaris)* in the presence and absence of the oomycete pathogen Aphanomyces euteiches. Biocontrol Sci. Technol. 27, 1123–44. 10.1080/09583157.2017.1376035

[B45] Pritsch C. Muehlbauer G. J. Bushnell W. R. Somers D. A. Vance C. P. (2000). Fungal development and induction of defense response genes during early infection of wheat spikes by *Fusarium graminearum*. Mol. Plant-Microbe Interact. 13, 159–69. 10.1094/MPMI.2000.13.2.159 10659706

[B46] Repo-Carrasco R. Espinoza C. Jacobsen S. E. (2003). Nutritional value and use of the andean crops quinoa (*Chenopodium quinoa*) and Kañiwa (*Chenopodium pallidicaule*). Food Rev. Int. 19, 179–89. 10.1081/FRI-12001888425794203

[B47] Robinson M. D. McCarthy D. J. Smyth G. K. (2010). edgeR: a Bioconductor package for differential expression analysis of digital gene expression data. Bioinformatics. 26, 139–40. 10.1093/bioinformatics/btp616 19910308 PMC2796818

[B48] Rollano-Peñaloza O. M. Widell S. Mollinedo P. Rasmusson A. G. (2018). *Trichoderma harzianum* T-22 and BOL-12QD inhibit lateral root development of *Chenopodium quinoa* in axenic co-culture. Cogent Biol. 4, 1–12. 10.1080/23312025.2018.1530493

[B49] Ruiz K. B. Biondi S. Oses R. Acuña-Rodríguez I. S. Antognoni F. Martinez-Mosqueira E. A. (2014). Quinoa biodiversity and sustainability for food security under climate change. A review. Agron. Sustain. Dev. 34, 349–59. 10.1007/s13593-013-0195-0

[B50] Salas-Marina M. A. Silva-Flores M. A. Uresti-Rivera E. E. Castro-Longoria E. Herrera-Estrella A. Casas-Flores S. (2011). Colonization of *Arabidopsis* roots by *Trichoderma atroviride* promotes growth and enhances systemic disease resistance through jasmonic acid/ethylene and salicylic acid pathways. Eur. J. Plant Pathol. 131, 15–26. 10.1007/s10658-011-9782-6

[B51] Saravanakumar K. Wang S. Dou K. Lu Z. Chen J. (2018). Yeast two-hybrid and label-free proteomics based screening of maize root receptor to cellulase of *Trichoderma harzianum*. Physiol. Mol. Plant Pathol. 104, 86–94. 10.1016/j.pmpp.2018.10.002

[B52] Schmidt J. Dotson B. R. Schmiderer L. van Tour A. Kumar B. Marttila S. (2020). Substrate and plant genotype strongly influence the growth and gene expression response to *Trichoderma afroharzianum* T22 in sugar Beet. Plants. 9, 1005. 10.3390/plants9081005 32784636 PMC7464433

[B53] Takahashi A. Casais C. Ichimura K. Shirasu K. (2003). HSP90 interacts with RAR1 and SGT1 and is essential for RPS2-mediated disease resistance in *Arabidopsis*. Proc. Natl. Acad. Sci. U.S.A. 100, 11777–82. 10.1073/pnas.2033934100 14504384 PMC208834

[B54] Tian T. Liu Y. Yan H. You Q. Du Y. i. X. (2017). agriGO v2.0: a GO analysis toolkit for the agricultural community, 2017 update. Nucleic Acids Res. 45, W122–W9. 10.1093/nar/gkx382 28472432 PMC5793732

[B55] Treutter D. (2006). Significance of flavonoids in plant resistance: a review. Environ. Chem. Lett. 4, 147–57. 10.1007/s10311-006-0068-8

[B56] Tucci M. Ruocco M. Masi D. e. De Palma L. Lorito M. (2011). The beneficial effect of *Trichoderma* spp. on tomato is modulated by the plant genotype. Mol. Plant Pathol. 12, 341–54. 10.1111/j.1364-3703.2010.00674.x 21453429 PMC6640367

[B57] Vega-Gálvez A. Miranda M. Vergara J. Uribe E. Puente L. Martínez E. A. (2010). Nutrition facts and functional potential of quinoa (*Chenopodium quinoa* Willd.), an ancient Andean grain: a review. J. Sci. Food Agric. 90, 2541–7. 10.1002/jsfa.4158 20814881

[B58] Venisse J-. S. Malnoy M. Faize M. Paulin J-. P. Brisset M-. N. (2002). Modulation of defense responses of *Malus* spp. during compatible and incompatible interactions with Erwinia amylovora. Mol. Plant-Microbe Interact. 15, 1204–12. 10.1094/MPMI.2002.15.12.1204 12481992

[B59] Verma M. Brar S. K. Tyagi R. D. Surampalli R. Y. Valéro J. R. (2007). Antagonistic fungi, *Trichoderma* spp.: Panoply of biological control. Biochem. Eng. J. 37, 1–20. 10.1016/j.bej.2007.05.012

[B60] Vos C. M. F. Cremer D. e. Cammue K. De Coninck B. P. A. (2015). The B. toolbox of *Trichoderma* spp. in the biocontrol of Botrytis cinerea disease. Mol. Plant Pathol. 16, 400–12. 10.1111/mpp.12189 25171761 PMC6638538

[B61] Wallström S. V. Aidemark M. Escobar M. A. Rasmusson A. G. (2012). An alternatively spliced domain of the *NDC1* NAD(P)H dehydrogenase gene strongly influences the expression of the *ACTIN2* reference gene in *Arabidopsis thaliana*. Plant Sci. 183, 190–6. 10.1016/j.plantsci.2011.08.011 22195593

[B62] Yasui Y. Hirakawa H. Oikawa T. Toyoshima M. Matsuzaki C. Ueno M. (2016a). Draft genome sequence of an inbred line of *Chenopodium quinoa*, an allotetraploid crop with great environmental adaptability and outstanding nutritional properties. DNA Res. 23, 535–46. 10.1093/dnares/dsw037 27458999 PMC5144677

[B63] Yasui Y. Hirakawa H. Oikawa T. Toyoshima M. Matsuzaki C. Ueno M. (2016b). Draft genome sequence of an inbred line of *Chenopodium quinoa* an allotetraploid crop with great environmental adaptability and outstanding nutritional properties. DNA Res. 2016, dsw037. 10.1093./dnares/dsw037 27458999 PMC5144677

[B64] Yedidia I. Benhamou N. Chet I. (1999). Induction of defense responses in cucumber plants (*Cucumis sativus L*.) by the biocontrol agent *Trichoderma harzianum*. Appl. Environ. Microbiol. 65, 1061–70. 10.1128/AEM.65.3.1061-1070.1999 10049864 PMC91145

[B65] Zhang X-. C. Millet Y. A. Cheng Z. Bush J. Ausubel F. M. (2015). Jasmonate signalling in *Arabidopsis* involves SGT1b–HSP70–HSP90 chaperone complexes. Nature Plants. 1, 15049. 10.1038/nplants.2015.49 27054042 PMC4819967

[B66] Zimmermann G. Bäumlein H. Mock H-. P. Himmelbach A. Schweizer P. (2006). The multigene family encoding germin-like proteins of barley. Regulation and function in basal host resistance. Plant Physiol. 142, 181–92. 10.1104/pp.106.083824 16844832 PMC1557593

[B67] Zurita-Silva A. Fuentes F. Zamora P. Jacobsen S-. E. Schwember A. R. (2014). Breeding quinoa (*Chenopodium quinoa* Willd.): potential and perspectives. Mol. Breeding. 34, 13–30. 10.1007/s11032-014-0023-5

