# Supplementary material for: Transcriptomic Analysis of Quinoa Reveals a Group of Germin-Like Proteins Induced by Trichoderma
Source: Front Fungal Biol. 2021 Dec 1;2:768648. doi: 10.3389/ffunb.2021.768648 (PMC10512214; doi:10.3389/ffunb.2021.768648)
Supplement: Supplementary file 1 [file Data_Sheet_1.PDF]

Table S1. Quinoa genes significantly upregulated in the cultivar Kurmi but not in Real

The table shows genes that were significantly upregulated in the Kurmi cultivar when treated with either BOL-12 or T22. The family of GLPs is highlighted in light green. The flavonoid biosynthetic pathway is highlighted in light purple. The numbers indicate averages of CPM values for each treatment (n = 3).

Quinoa gene name	Quinoa gene code	Gene description[a]	Kurmi			Real		
			Ctrl	B12	T22	Ctrl	B12	T22
CqGLP-7	AUR62025241	Plant defensin	0,4	13,1	4,4	14,6	40,4	30,8
PR-10	AUR62025514	Major allergen Api g isoallergen 2	0,3	11,5	1,4	3,2	7,3	8,3
CqGLP-17	AUR62025235	Plant defensin	0,6	13,5	3,2	13,1	24,8	26,3
CYP81E8	AUR62038793	Cytochrome P450 81E8	1,5	31,6	6,8	20,2	50,0	29,3
COMT1	AUR62005985	Caffeic acid 3-O-methyltransferase	0,9	17,2	3,4	13,6	23,4	21,4
CHS	AUR62013677	Chalcone synthase	3,0	51,3	12,2	41,9	83,5	75,1
CqGLP-10	AUR62025242	Plant defensin	2,9	39,2	18,2	47,6	108,5	91,6
CqGLP-2	AUR62025224	Plant defensin	1,9	22,0	10,8	23,7	49,3	47,5
CqGLP-9	AUR62028952	Plant defensin	0,6	6,6	4,6	13,7	28,2	22,4
STPS	AUR62038591	Probable sesquiterpene synthase	6,8	75,3	26,4	81,3	151,1	97,6
CqGLP-6	AUR62025237	Plant defensin	2,1	22,9	12,2	36,5	89,6	61,8
NN	AUR62026572	Protein of unknown function	0,5	4,9	1,3	8,4	14,1	14,6
CqGLP-14	AUR62025218	Plant defensin	1,9	19,8	15,2	32,1	71,6	73,7
RCE1	AUR62043544	NEDD8-conjugating enzyme Ubc12	0,7	7,1	3,1	0,4	0,9	0,7
CqGLP-15	AUR62025225	Plant defensin	3,5	34,8	26,6	41,5	101,4	91,3
CqGLP-18	AUR62025236	Plant defensin	4,4	38,4	22,3	87,0	136,1	153,9
CqGLP-21	AUR62028956	Plant defensin	0,6	5,0	2,4	9,0	11,5	13,1
Chit1	AUR62033302	Chitotriosidase-1	0,3	2,8	1,9	1,4	3,3	3,3
CqGLP-12	AUR62028948	Plant defensin	3,6	28,8	15,2	31,5	63,5	53,5
D4H	AUR62028217	Deacetoxyvindoline 4-hydroxylase	11,9	74,2	39,1	87,5	152,4	123,8
cmbl	AUR62002636	Carboxymethylenebutenolidase homolog	1,5	8,7	7,5	32,8	33,6	41,9
CISZOG1	AUR62026077	Cis-zeatin O-glucosyltransferase 1	1,9	11,2	4,3	3,7	11,0	9,8
NN	AUR62028130	At1g58390 Probable disease resistance	0,6	3,1	2,7	5,1	3,1	2,6
CqGLP-3	AUR62025232	Plant defensin	0,4	2,3	2,4	8,2	13,1	15,9
NN	AUR62040548	Protein of unknown function	0,4	2,1	1,2	2,8	3,4	2,5
CHI3	AUR62030612	Probable chalcone-flavonone isomerase 3	6,9	34,4	18,0	26,1	54,6	47,6

CqGLP-13	AUR62025219	Plant defensin	0,7	3,7	3,4	6,0	12,2	12,6
CqGLP-1	AUR62025223	Plant defensin	7,6	34,7	29,4	40,0	93,6	77,0
CqPR4	AUR62001001	Pathogenesis-related protein P2	6,9	30,0	15,6	38,7	68,2	54,4
UGT73C5	AUR62024488	UDP-glycosyltransferase 73C5	1,7	7,6	3,9	5,9	13,0	9,2
NN	AUR62037762	Protein of unknown function	0,5	2,1	1,7	5,1	4,0	7,0
CqGLP-19	AUR62028958	Plant defensin	10,7	41,9	43,3	67,0	144,2	118,9
CqGLP-20	AUR62040806	Plant defensin	3,9	15,0	12,1	59,9	102,1	78,5
BURP5	AUR62011666	BURP domain-containing protein 5	7,8	30,0	21,7	33,3	58,5	60,1
NN	AUR62016052	Protein of unknown function	0,3	1,4	1,9	1,3	0,5	0,3
CG5412	AUR62019002	UPF0483 protein CG5412	2,2	8,0	5,0	7,2	10,7	9,5
CqGLP-16	AUR62025234	Plant defensin	16,9	56,5	41,9	75,5	132,7	130,1
NN	AUR62011182	Protein of unknown function	0,9	2,9	2,6	2,3	1,6	2,1
FL	AUR62014672	Flavonol synthase/flavanone 3-hydroxylase	2,2	6,1	6,2	7,8	11,4	12,5
NN	AUR62033227	At5g57670	1,8	4,8	4,0	2,5	3,0	3,2
PER8	AUR62007739	Peroxidase 8	1,5	3,9	3,2	6,4	4,5	4,1
NN	AUR62020367	Protein of unknown function	1,5	3,9	4,0	2,9	1,8	2,5
CHI	AUR62020547	Chalcone-flavonone isomerase	27,8	68,2	43,8	63,1	100,2	100,1
RR9	AUR62026868	Response regulator ORR9	2,4	5,9	9,2	6,4	4,0	6,1
NN	AUR62027119	Protein of unknown function	5,6	13,3	9,6	29,3	38,4	52,5
NN	AUR62041108	Protein of unknown function	3,6	8,5	7,4	8,8	6,7	9,4
NN	AUR62001977	GDSL esterase/lipase At2g23540	12,2	26,0	23,6	46,9	73,2	96,6
NN	AUR62026150	Protein of unknown function	4,8	10,1	8,6	14,5	14,9	20,7
NN	AUR62003568	Protein of unknown function	32,4	67,1	57,7	74,2	113,8	113,6
NN	AUR62006030	Protein of unknown function	3,9	7,7	13,0	4,9	3,4	3,6
CHI3	AUR62024932	Probable chalcone-flavonone isomerase 3	39,4	78,6	61,5	66,8	99,1	104,7
CqRSLP 2	AUR62031352	Os03g0733400 RICESLEEPER 2	11,1	21,7	19,2	22,4	23,3	35,9
SAUR71	AUR62001328	Auxin-responsive protein SAUR71	5,1	9,9	8,3	10,0	6,4	8,3
SPAC644.07	AUR62018315	Probable mitochondrial chaperone bcs1	12,2	23,1	18,0	13,7	24,9	22,5
CAR4	AUR62018204	Protein C2-DOMAIN ABA-RELATED 4	7,7	14,4	13,6	20,8	21,6	27,9
NN	AUR62023337	Protein of unknown function	24,4	42,2	36,1	58,0	38,3	56,1
GDPD1	AUR62029437	GDPD1	11,1	19,0	25,0	27,0	22,7	26,9
CHI	AUR62015655	Chalcone-flavonone isomerase	75,8	126,7	102,3	102,7	132,9	133,2
CDC2	AUR62033172	Cell division control protein 2 homolog	12,6	20,8	23,4	19,3	17,7	29,3

[a] Jarvis et al., 2017

Table S2. Quinoa genes significantly downregulated in the cultivar Kurmi but not in Real

Genes observed to be significantly downregulated in the Kurmi cultivar when treated with either BOL-12 or T22 are included. The numbers indicate average of CPM values for every treatment (n = 3). CPM of reference genes are showed at the bottom for transparency.

Quinoa gene name	Quinoa gene code	Gene description[a]	Kurmi			Real		
			Ctrl	B12	T22	Ctrl	B12	T22
CDC20-1	AUR62028149	Cell division cycle 20.1	77,3	50,1	36,1	45,8	72,7	32,3
CDC20-1	AUR62005915	Cell division cycle 20.1 cofactor of APC complex	52,9	34,2	24,4	32,6	45,6	23,0
OLP	AUR62001811	Osmotin-like protein	34,3	21,2	15,5	20,9	34,5	17,8
NN	AUR62036106	Protein of unknown function	70,0	43,3	40,0	48,0	74,6	36,9
NN	AUR62009169	Protein of unknown function	34,2	20,0	15,9	13,0	30,7	13,3
SWEET6B	AUR62029552	Bidirectional sugar transporter SWEET6b	28,7	16,5	15,2	16,7	21,5	18,7
PG	AUR62030002	Polygalacturonase At1g48100	28,3	15,4	9,7	18,8	16,9	14,7
WAG1	AUR62019653	Serine/threonine-protein kinase WAG1	33,0	17,3	17,6	14,9	18,3	15,2
WAG1	AUR62013960	Serine/threonine-protein kinase WAG1	13,1	6,8	6,0	6,9	7,8	4,3
LBD41	AUR62023311	LOB domain-containing protein 41	7,8	3,7	1,9	2,2	5,2	3,0
PCKA	AUR62005878	Phosphoenolpyruvate carboxykinase [ATP]	27,9	13,1	13,6	4,2	5,0	4,3
LECRKS7	AUR62028107	Probable L-type lectin-domain containing receptor kinase S.7	5,5	2,4	3,0	2,7	3,2	3,4
NN	AUR62004646	Protein of unknown function	8,8	3,4	4,1	3,3	0,9	2,8
CqEGlu	AUR62043172	Glucan endo-beta-glucosidase	3,3	1,0	0,9	0,4	0,5	1,0
MHB1	AUR62011380	Non-symbiotic hemoglobin 1	10,4	2,9	2,9	1,5	1,9	2,4
PG	AUR62002459	Polygalacturonase	6,1	1,3	1,7	1,5	1,9	1,1
CqAct2A	AUR62014374	CqActin2 Ref Gene	806,8	787,1	539,6	710,1	724,8	687,1
CqAct2B	AUR62019116	CqActin2 Ref Gene	812,1	692,9	527,3	655,8	661,4	612,3
CqAct2C	AUR62014579	CqActin2 Ref Gene	81,8	70,0	60,9	74,7	69,6	57,0
CqAct2D	AUR62039382	CqActin2 Ref Gene	141,1	131,8	112,7	130,0	113,5	91,7
CqMon1A	AUR62020295	CqMon1 Ref Gene	45,6	41,0	41,6	38,3	39,6	35,2
CqMon1B	AUR62037705	CqMon1 Ref Gene	35,2	36,3	33,2	26,8	28,3	27,0

[a] Jarvis et al., 2017

Table S3. Quinoa genes significantly up- and downregulated in the cultivar Real but not in Kurmi.

Genes shown were significantly and consistently upregulated or downregulated in the Real cultivar when treated with either BOL-12 or T22. Highlighted in green we can observe a family of chitinases. Light purple highlights WRKY genes and orange highlights ethylene-responsive genes.

Quinoa gene name	Quinoa gene code	Gene description
OPR3	AUR62025200	12-oxophytodienoate reductase 3
DODA1	AUR62012347	2C5-DOPA dioxygenase extradiol 1
KCS1	AUR62022664	3-ketoacyl-CoA synthase 1
KCS2	AUR62006329	3-ketoacyl-CoA synthase 2
ABCG6	AUR62025538	ABC transporter G family member 6
CYP74A	AUR62040103	Allene oxide synthase chloroplastic
nep1	AUR62038206	Aspartic proteinase nepenthesin-1
nep2	AUR62015131	Aspartic proteinase nepenthesin-2
BAM3	AUR62018803	Beta-amylase 3%2C chloroplastic
CAMBP25	AUR62018430	Calmodulin-binding protein 25
Cht4	AUR62027403	Chitinase 4
ATX1	AUR62000774	Copper transport protein ATX1
CYP86A8	AUR62004370	Cytochrome P450 86A8
CYP86A8	AUR62022579	Cytochrome P450 86A8
ATL6	AUR62000873	E3 ubiquitin-protein ligase ATL6
HOS3	AUR62008021	Elongation of fatty acids protein 3-like
EPHX4	AUR62007573	Epoxide hydrolase 4
ERF4	AUR62017052	Ethylene-responsive transcription factor 4
ERF5	AUR62018880	Ethylene-responsive transcription factor 5
ERF9	AUR62014855	Ethylene-responsive transcription factor 9
ERF017	AUR62016128	Ethylene-responsive transcription factor ERF017
EXO70A1	AUR62008535	Exocyst complex component EXO70A1
EXO70B1	AUR62009665	Exocyst complex component EXO70B1
XLG1	AUR62016663	Extra-large guanine nucleotide-binding protein 1
SKIP30	AUR62028597	F-box/kelch-repeat protein SKIP30
CYP75B1	AUR62008515	Flavonoid 3'-monooxygenase

CYP75B2	AUR62021575	Flavonoid 3'-monooxygenase
CYP75A1	AUR62009892	Flavonoid 5'-hydroxylase 1
FH8	AUR62026311	Formin-like protein 8
At5g24080	AUR62021884	G-type lectin S-receptor-like serine/threonine-protein kinase At5g24080
GATA5	AUR62010402	GATA transcription factor 5
At5g13200	AUR62005695	GEM-like protein 5
GA2OX2	AUR62011753	Gibberellin 2-beta-dioxygenase 2
At4g29360	AUR62020357	Glucan endo-3-beta-glucosidase 12
GPAT4	AUR62004368	Glycerol-3-phosphate 2-O-acyltransferase 4
GPAT4	AUR62022577	Glycerol-3-phosphate 2-O-acyltransferase 4
GPAT5	AUR62012122	Glycerol-3-phosphate acyltransferase 5
GPAT5	AUR62040714	Glycerol-3-phosphate acyltransferase 5
HSF24	AUR62001930	Heat shock factor protein HSF24
HHP1	AUR62013296	Heptahelical transmembrane protein 1
LAC12	AUR62012314	Laccase-12
LAC12	AUR62022954	Laccase-12
LHT1	AUR62023955	Lysine histidine transporter 1
LHT1	AUR62031750	Lysine histidine transporter 1
1MMP	AUR62007090	Metalloendoproteinase 1-MMP
MLP43	AUR62043142	MLP-like protein 43
MYB4	AUR62015573	Myb-related protein Myb4
mhkB	AUR62011395	Myosin heavy chain kinase B
CHLN	AUR62000396	Nicotianamine synthase
PAE2	AUR62002521	Pectin acetylesterase 2
PER24	AUR62034604	Peroxidase 24
PER24	AUR62036667	Peroxidase 24
At4g16820	AUR62018869	Phospholipase A1-Ibeta2%2C chloroplastic
At1g06800	AUR62008926	Phospholipase A1-Igamma1 chloroplastic
PGIP2	AUR62024807	Polygalacturonase inhibitor 2
MJ0612	AUR62013277	Probable arogenate/prephenate dehydrogenase
CML36	AUR62026154	Probable calcium-binding protein CML36
CAF1-11	AUR62020248	Probable CCR4-associated factor 1 homolog 11
CAD6	AUR62018276	Probable cinnamyl alcohol dehydrogenase 6

At1g62630	AUR62025629	Probable disease resistance protein At1g62630
GATL1	AUR62016655	Probable galacturonosyltransferase-like 1
At1g74360	AUR62021617	Probable LRR receptor-like serine/threonine-protein kinase
RKF3	AUR62006561	Probable LRR receptor-like serine/threonine-protein kinase RKF3
At2g30020	AUR62020159	Probable protein phosphatase 2C 25
WRKY11	AUR62020288	Probable WRKY transcription factor 11
WRKY23	AUR62039260	Probable WRKY transcription factor 23
WRKY33	AUR62026343	Probable WRKY transcription factor 33
WRKY40	AUR62030836	Probable WRKY transcription factor 40
WRKY41	AUR62010821	Probable WRKY transcription factor 41
WRKY41	AUR62019820	Probable WRKY transcription factor 41
WRKY42	AUR62021917	Probable WRKY transcription factor 42
WRKY57	AUR62003119	Probable WRKY transcription factor 57
WRKY70	AUR62029778	Probable WRKY transcription factor 70
XTH23	AUR62027741	Probable xyloglucan endotransglucosylase/hydrolase protein XTH23
EXO	AUR62027794	Protein EXORDIUM
EXL2	AUR62027796	Protein EXORDIUM-like 2
LYK5	AUR62043269	Protein LYK5
SARD1	AUR62033803	Protein SAR DEFICIENT 1
YLS9	AUR62037049	Protein YLS9
YLS9	AUR62044049	Protein YLS9
RGA4	AUR62033818	Putative disease resistance protein RGA4
RPPL1	AUR62007274	Putative disease resistance RPP13-like protein 1
PGSIP7	AUR62042795	Putative glucuronosyltransferase PGSIP7
PERK11	AUR62008808	Putative proline-rich receptor-like protein kinase PERK11
ATL2	AUR62015272	RING-H2 finger protein ATL2
ATL2	AUR62018069	RING-H2 finger protein ATL2
SCL13	AUR62021833	Scarecrow-like protein 13
SCL5	AUR62009552	Scarecrow-like protein 5
At3g07070	AUR62020037	Serine/threonine-protein kinase At3g07070
At5g01020	AUR62030734	Serine/threonine-protein kinase At5g01020
SDR2a	AUR62009713	Short-chain dehydrogenase reductase 2a
HSP23	AUR62039962	Small heat shock protein chloroplastic

SYP132	AUR62036357	Syntaxin-132
MYB44	AUR62010308	Transcription factor MYB44
RAX3	AUR62012493	Transcription factor RAX3
TYDC2	AUR62025133	Tyrosine/DOPA decarboxylase 2
PUB26	AUR62010239	U-box domain-containing protein 26
PUB4	AUR62003956	U-box domain-containing protein 4
UGT87A2	AUR62001891	UDP-glycosyltransferase 87A2
At3g50280	AUR62018684	Uncharacterized acetyltransferase At3g50280
At3g28850	AUR62034387	Uncharacterized protein At3g28850
SAP7	AUR62017364	Zinc finger A20 and AN1 domain-containing stress-associated protein 7
ZAT12	AUR62001834	Zinc finger protein ZAT12
ZAT12	AUR62009622	Zinc finger protein ZAT12
ZAT12	AUR62038383	Zinc finger protein ZAT12
NN	AUR62034001	5-epi-aristolochene synthase 3
CqChit1	AUR62027407	Acidic endochitinase
NN	AUR62010867	B2 protein
NN	AUR62019730	Basic 7S globulin 2
NN	AUR62011155	Cucumber peeling cupredoxin
CqChit1	AUR62019040	Endochitinase
NN	AUR62039951	Hydrophobic seed protein
NN	AUR62002452	Protein of unknown function
NN	AUR62003653	Protein of unknown function
NN	AUR62007952	Protein of unknown function
NN	AUR62008152	Protein of unknown function
NN	AUR62008676	Protein of unknown function
NN	AUR62009687	Protein of unknown function
NN	AUR62010274	Protein of unknown function
NN	AUR62011712	Protein of unknown function
NN	AUR62012437	Protein of unknown function
NN	AUR62012922	Protein of unknown function
NN	AUR62012928	Protein of unknown function
NN	AUR62016627	Protein of unknown function
NN	AUR62017310	Protein of unknown function

NN	AUR62018946	Protein of unknown function
NN	AUR62020224	Protein of unknown function
NN	AUR62021162	Protein of unknown function
NN	AUR62022193	Protein of unknown function
NN	AUR62023931	Protein of unknown function
NN	AUR62024933	Protein of unknown function
NN	AUR62025086	Protein of unknown function
NN	AUR62025343	Protein of unknown function
NN	AUR62027173	Protein of unknown function
NN	AUR62027736	Protein of unknown function
NN	AUR62031561	Protein of unknown function
NN	AUR62032323	Protein of unknown function
NN	AUR62033664	Protein of unknown function
NN	AUR62034721	Protein of unknown function
NN	AUR62038186	Protein of unknown function
NN	AUR62040531	Thaumatin-like protein 1

Table S4. Singular enrichment analysis of differentially expressed genes in quinoa roots treated with *Trichoderma*

For each quinoa-*Trichoderma* interaction, quinoa genes differentially expressed (DE) were annotated for Gene Ontology with Argot2 and then analyzed for singular enrichment analysis with AgriGO2. Stress-related GO-term are highlighted in grey and cell wall-related terms in orange.

Interaction	GO term	Description	DE genes	Total genes	p-value	FDR
Kurmi and T22	-	None	-	-	-	-
Kurmi and BOL-12	GO:0006952	defense response	6	108	5,8E-05	0,01
	GO:0009607	response to biotic stimulus	5	83	1,6E-04	0,02
Real and BOL-12	GO:0006073	cellular glucan metabolic process	6	117	2,9E-05	0,00
	GO:0044042	glucan metabolic process	6	117	2,9E-05	0,00
	GO:0044264	cellular polysaccharide metabolic process	6	117	2,9E-05	0,00
	GO:0005976	polysaccharide metabolic process	6	140	7,8E-05	0,01
	GO:0044262	cellular carbohydrate metabolic process	6	174	2,6E-04	0,02
	GO:0043043	peptide biosynthetic process	10	599	1,0E-03	0,03
	GO:0034645	cellular macromolecule biosynthetic process	22	1977	7,3E-04	0,03
	GO:0044271	cellular nitrogen compound biosynthetic process	21	1907	1,0E-03	0,03
	GO:0071554	cell wall organization or biogenesis	5	144	7,9E-04	0,03
	GO:0043604	amide biosynthetic process	10	599	1,0E-03	0,03
	GO:0009059	macromolecule biosynthetic process	22	1979	7,4E-04	0,03
	GO:0006412	translation	10	591	9,3E-04	0,03
	GO:0006518	peptide metabolic process	10	613	1,2E-03	0,04
	GO:0043603	cellular amide metabolic process	10	622	1,4E-03	0,04
Real and T-22	GO:0080090	regulation of primary metabolic process	80	983	4,9E-12	0,00
	GO:0060255	regulation of macromolecule metabolic process	81	1001	4,9E-12	0,00
	GO:2001141	regulation of RNA biosynthetic process	78	933	2,4E-12	0,00

GO:0009889	regulation of biosynthetic process	79	961	4,0E-12	0,00
GO:0006355	regulation of transcription, DNA-templated	78	933	2,4E-12	0,00
GO:0010556	regulation of macromolecule biosynthetic process	79	961	4,0E-12	0,00
GO:1903506	regulation of nucleic acid-templated transcription	78	933	2,4E-12	0,00
GO:0051252	regulation of RNA metabolic process	78	935	2,6E-12	0,00
GO:0031326	regulation of cellular biosynthetic process	79	961	4,0E-12	0,00
GO:0031323	regulation of cellular metabolic process	80	983	4,9E-12	0,00
GO:2000112	regulation of cellular macromolecule biosynthetic process	79	961	4,0E-12	0,00
GO:0010468	regulation of gene expression	80	974	3,1E-12	0,00
GO:0019219	regulation of nucleobase-containing compound metabolic process	78	947	4,9E-12	0,00
GO:0019222	regulation of metabolic process	81	1003	5,4E-12	0,00
GO:0051171	regulation of nitrogen compound metabolic process	79	968	5,7E-12	0,00
GO:0016567	protein ubiquitination	19	84	5,0E-11	0,00
GO:0032446	protein modification by small protein conjugation	19	86	7,7E-11	0,00
GO:0036211	protein modification process	133	2135	3,0E-10	0,00
GO:0006464	cellular protein modification process	133	2135	3,0E-10	0,00
GO:0044260	cellular macromolecule metabolic process	251	4736	5,5E-10	0,00
GO:0043412	macromolecule modification	134	2195	8,6E-10	0,00
GO:0097659	nucleic acid-templated transcription	78	1126	1,1E-08	0,00
GO:0006351	transcription, DNA-templated	78	1126	1,1E-08	0,00
GO:0032774	RNA biosynthetic process	78	1130	1,2E-08	0,00
GO:0019438	aromatic compound biosynthetic process	86	1293	1,4E-08	0,00
GO:0043170	macromolecule metabolic process	266	5308	2,0E-08	0,00
GO:1901362	organic cyclic compound biosynthetic process	88	1347	2,1E-08	0,00
GO:0034654	nucleobase-containing compound biosynthetic process	80	1208	4,6E-08	0,00

GO:0044237	cellular metabolic process	288	5911	6,7E-08	0,00
GO:0018130	heterocycle biosynthetic process	84	1308	8,3E-08	0,00
GO:0006468	protein phosphorylation	102	1739	3,2E-07	0,00
GO:0070647	protein modification by small protein conjugation or removal	19	141	3,7E-07	0,00
GO:0016310	phosphorylation	102	1845	3,8E-06	0,00
GO:0050789	regulation of biological process	88	1533	3,8E-06	0,00
GO:0050794	regulation of cellular process	87	1511	3,8E-06	0,00
GO:0044267	cellular protein metabolic process	146	2859	4,1E-06	0,00
GO:0008152	metabolic process	438	10087	1,0E-05	0,00
GO:0065007	biological regulation	89	1610	1,3E-05	0,00
GO:0005976	polysaccharide metabolic process	16	140	2,4E-05	0,00
GO:0071704	organic substance metabolic process	310	7010	2,8E-05	0,00
GO:0006796	phosphate-containing compound metabolic process	109	2110	3,0E-05	0,00
GO:0006793	phosphorus metabolic process	109	2117	3,4E-05	0,00
GO:0006073	cellular glucan metabolic process	14	117	4,5E-05	0,00
GO:0044264	cellular polysaccharide metabolic process	14	117	4,5E-05	0,00
GO:0044042	glucan metabolic process	14	117	4,5E-05	0,00
GO:0009987	cellular process	310	7066	4,6E-05	0,00
GO:0046348	amino sugar catabolic process	6	22	6,9E-05	0,00
GO:0016998	cell wall macromolecule catabolic process	6	22	6,9E-05	0,00
GO:1901071	glucosamine-containing compound metabolic process	6	22	6,9E-05	0,00
GO:0044036	cell wall macromolecule metabolic process	6	22	6,9E-05	0,00
GO:1901072	glucosamine-containing compound catabolic process	6	22	6,9E-05	0,00
GO:0006026	aminoglycan catabolic process	6	22	6,9E-05	0,00
GO:0006030	chitin metabolic process	6	22	6,9E-05	0,00

GO:0006032	chitin catabolic process	6	22	6,9E-05	0,00
GO:0006040	amino sugar metabolic process	6	22	6,9E-05	0,00
GO:0044271	cellular nitrogen compound biosynthetic process	98	1907	8,1E-05	0,00
GO:0016070	RNA metabolic process	83	1575	1,1E-04	0,00
GO:0044238	primary metabolic process	292	6719	1,1E-04	0,00
GO:0019538	protein metabolic process	158	3387	1,4E-04	0,00
GO:1901136	carbohydrate derivative catabolic process	7	35	1,5E-04	0,00
GO:0006022	aminoglycan metabolic process	6	30	4,4E-04	0,01
GO:0044249	cellular biosynthetic process	117	2500	6,5E-04	0,01
GO:1901576	organic substance biosynthetic process	117	2516	8,0E-04	0,01
GO:0034645	cellular macromolecule biosynthetic process	95	1977	8,2E-04	0,01
GO:0009059	macromolecule biosynthetic process	95	1979	8,5E-04	0,01
GO:0032501	multicellular organismal process	11	105	8,9E-04	0,01
GO:0044262	cellular carbohydrate metabolic process	15	174	9,3E-04	0,01
GO:0006950	response to stress	38	650	1,1E-03	0,01
GO:0010467	gene expression	96	2025	1,2E-03	0,02
GO:0006633	fatty acid biosynthetic process	9	82	1,8E-03	0,02
GO:0016053	organic acid biosynthetic process	14	173	2,4E-03	0,03
GO:0072330	monocarboxylic acid biosynthetic process	9	86	2,5E-03	0,03
GO:0044283	small molecule biosynthetic process	16	216	3,1E-03	0,04
GO:0055114	oxidation-reduction process	93	2040	3,8E-03	0,04
GO:0044703	multi-organism reproductive process	8	75	3,8E-03	0,04
GO:0044706	multi-multicellular organism process	8	75	3,8E-03	0,04
GO:0009875	pollen-pistil interaction	8	75	3,8E-03	0,04
GO:0008037	cell recognition	8	75	3,8E-03	0,04

GO:0048544	recognition of pollen	8	75	3,8E-03	0,04
GO:0009856	pollination	8	75	3,8E-03	0,04
GO:0006979	response to oxidative stress	18	266	4,6E-03	0,05

Table S5. Gene expression assessed by RNA-seq and qRT-PCR.

RNA from quinoa roots treated with *Trichoderma* or mock treated (12 hpi) were analysed by RNA-seq and qRT-PCR in order to determine the correlation of expression levels. Fold change was determined by comparing samples treated with each *Trichoderma* strain against the mock-treated control.

Method	Gene	Code	Kurmi		Real	
			BOL-12	T22	BOL-12	T22
			FC(log2)	FC(log2)	FC(log2)	FC(log2)
qRT-PCR*	*CqCat2*	*AUR62040809*	0,78	0,71	1,09	1,35
	CqEDS	*AUR62001194*	0,88	0,79	0,82	0,62
	CqGLP1	*AUR62025223*	4,14	4,10	1,71	1,26
	CqGLP10	*AUR62025242*	14,60	8,84	2,00	1,79
	CqHSP90	*AUR62031424*	1,39	2,08	1,28	2,44
	CqMon1	*AUR62020295*	1,01	1,01	0,97	1,02
	CqMyc2	*AUR62018713*	1,02	1,26	1,07	1,86
	CqPER39	*AUR62034603*	1,14	1,56	0,54	0,61
	CqPR1C	*AUR62027040*	0,91	1,24	0,32	0,50
	CqRAP2.3	*AUR62018057*	0,76	0,73	0,74	0,90
	CqW33	*AUR62006298*	2,33	3,52	2,36	4,35
RNA-seq	*CqCat2*	*AUR62040809*	0,78	0,60	1,31	1,51
	CqEDS	*AUR62001194*	0,93	1,07	0,98	0,94
	CqGLP1	*AUR62025223*	4,59	3,88	2,34	1,93
	CqGLP10	*AUR62025242*	13,51	6,26	2,28	1,92
	CqHSP90	*AUR62031424*	2,08	3,15	2,27	4,51
	CqMon1	*AUR62020295*	0,90	0,91	1,04	0,92
	CqMyc2	*AUR62018713*	0,89	0,90	1,94	2,99
	CqPER39	*AUR62034603*	0,84	1,94	0,59	0,47
	CqPR1C	*AUR62027040*	1,72	1,68	0,17	0,33
	CqRAP2.3	*AUR62018057*	0,82	0,44	1,43	1,36
	CqW33	*AUR62006298*	1,79	4,11	3,55	7,61
Correlation treatment dependent (R^2)			0,90	0,86	0,85	0,92
Correlation (R^2)			0,848			

Pearson correlation coefficient	0,921

* Normalized to *CqAct2*

Table S6. Gene expression in quinoa shoot and root at 36 hpi with *Trichoderma.*

Quinoa shoot and root samples were assessed by qRT-PCR after 36 h treatment with *Trichoderma* added to the roots. Gene expression was normalized to the CqAct2 reference gene. Fold change was determined by comparing samples treated with *Trichoderma* against mock-treated. Significant differences between treatment and control is highlighted in red. ND, not detected.

Tissue	Gene	Code	Kurmi				Real			
			BOL-12		T22		BOL-12		T22	
			FC(log2)	p-value	FC(log2)	p-value	FC(log2)	p-value	FC(log2)	p-value
Shoot	*CqCat2*	*AUR62040809*	-0,04	0,97	-0,61	0,00	-0,21	0,33	0,40	0,29
	CqEDS	*AUR62001194*	0,44	0,53	0,27	0,54	0,03	0,95	0,16	0,72
	CqGLP1	*AUR62025223*	0,95	0,04	0,01	0,95	1,31	0,03	-0,14	0,57
	CqGLP10	*AUR62025242*	1,12	0,03	0,82	0,27	1,77	0,01	0,51	0,24
	CqHSP83a	*AUR62031424*	-0,02	0,93	1,17	0,01	-0,44	0,24	1,31	0,01
	CqMon1	*AUR62020295*	0,11	0,46	0,08	0,22	0,04	0,85	-0,24	0,30
	CqMyc2	*AUR62018713*	-0,02	0,88	0,19	0,21	0,04	0,86	-0,76	0,13
	CqPER39	*AUR62034603*	ND	-	ND	-	ND	-	ND	-
	CqPR1C	*AUR62027040*	ND	-	ND	-	ND	-	ND	-
	CqRAP2.3	*AUR62018057*	0,07	0,62	-0,55	0,30	-0,04	0,76	0,21	0,25
	CqW33	*AUR62006298*	0,18	0,60	1,41	0,00	0,63	0,04	0,67	0,43
Root	*CqCat2*	*AUR62040809*	-0,47	0,01	0,08	0,63	-0,43	0,10	-0,22	0,18
	CqEDS	*AUR62001194*	-0,19	0,20	1,31	0,05	-0,27	0,56	1,33	0,04
	CqGLP1	*AUR62025223*	0,63	0,29	1,41	0,14	1,02	0,13	1,55	0,09
	CqGLP10	*AUR62025242*	1,01	0,04	1,59	0,03	1,54	0,15	2,65	0,01
	CqHSP83a	*AUR62031424*	-0,70	0,01	-0,01	0,99	-0,53	0,03	-0,15	0,64
	CqMon1	*AUR62020295*	0,15	0,17	-0,18	0,61	-0,15	0,33	0,25	0,11
	CqMyc2	*AUR62018713*	-0,37	0,04	0,25	0,50	-0,07	0,83	0,24	0,66
	CqPER39	*AUR62034603*	0,30	0,54	0,22	0,67	-0,63	0,20	-1,37	0,02
	CqPR1C	*AUR62027040*	0,39	0,34	0,56	0,55	0,48	0,10	-0,55	0,39
	CqRAP2.3	*AUR62018057*	-0,70	0,01	-0,27	0,28	-0,59	0,26	0,12	0,59
	CqW33	*AUR62006298*	0,09	0,81	1,41	0,00	-0,20	0,61	0,60	0,03

Table S7. Primer sequences of quinoa genes analysed by qRT-PCR.

Forward and reverse primer sequences for qRT-PCR. Primer pairs were designed using Perlprimer aiming for exon-exon borders. *CqAct2* were used as reference genes for normalization of the mRNA abundances and were further verified by the *CqMon1* housekeeping gene.

Quinoa gene	*C. quinoa* gene code	Putative encoding gene description		Primer forward	Primer reverse	Product	PCR eff.
CqAct2	*AUR62014374*	*actin2*	Reference gene	TACCACAGGTATCGTGCTTGACTC	GATCACGTCCGGCAAGATCC	113 bp	1,89
CqMon1A[a]	*AUR62020295*	*monensin sensitivity 1*	Reference gene	AAGGATCATCTGACCATAAAGC	TCGTGTCAAGTTAGTTCGGG	145 bp	1,98
CqCat2	*AUR62040809*	*catalase 2*	Defense-related	CCAGGAGTGAGATATAGATCATGGG	CCCAAAGATTTATCCGCCTGAG	145 bp	2,03
CqEDS	*AUR62001194*	*enhanced disease susceptibility 1*	Defense-related	TTTGTCAGCTTGTTCATCGT	GTCCTGCATATCTTTCTTCCC	125 bp	1,96
CqPR1C	*AUR62027040*	*Pathogenesis-related protein 1C*	Defense-related	TGTTTCATTGTCATAACCCTAGCC	ACTGTATGTTCACAACACCCAC	117 bp	1,93
CqGLP1	*AUR62025223*	*Germin-like protein 1*	Defensin	GCATTACAACTCACTCCTACC	CTTCATCCGCATAACTTCCT	123 bp	1,93
CqGLP10	*AUR62025242*	*Germin-like protein 10*	Defensin	ATACCAACAACACCGCACAC	GTAAGTTCCCAGCAAATAAAGCAG	179 bp	1,97
CqHSP83	*AUR62031424*	*heat shock protein 83*	Stress-related	ATTCGGTGTTGGTTTCTACTC	CCAAGTATTCCAACTGATCTTCC	199 bp	1,92
CqMyc2	*AUR62018713*	*Myc2 transcription factor*	Defense-related	GGAAGTGAAGGAGACGAGAA	CAACCCAGCATACTCCGAAA	102 bp	2,00
CqWRKY33	*AUR62006298*	*WRKY DNA-binding protein 33*	Defense-related	TCCTTTACACCTGAGACATCCT	ACTGTTCTGTTACCATACCCTGAC	126 bp	1,93
CqPER39	*AUR62034603*	*Peroxidase 39*	Defense-related	TTGTGATTTGTAATGCAGGTGG	CTCGAGGGCAACTCTTATGG	149 bp	2,03
CqRAP2.3	*AUR62018057*	*Ethylene response factor related to AP 2.2*	Stress-related	ATGATGAGTATGGGATTCTACGG	CCCAAGCAAAGATTCAAGGT	122 bp	2,01

[a] The primer pair matches 100% with the *CqMon1B* (AUR62037705) gene sequence which shares 95% nucleotide sequence identity with *CqMon1A*.

Table S8. Quinoa GLPs significantly upregulated upon treatment with *Trichoderma*.

Phytozome and NCBI codes for the germin-like proteins significantly upregulated by *Trichoderma* in the Kurmi cultivar. These GLPs belong to a quinoa-specific clade (Figure 6).

Gene name	Phytozome code	NCBI code	Peptide size (aa)
CqGLP-1*	AUR62025223	XP_021750106.1	208
CqGLP-2	AUR62025224	XP_021750088.1	208
CqGLP-3	AUR62025232	XP_021750260.1	208
CqGLP-6	AUR62025237	XP_021750078.1	208
CqGLP-7	AUR62025241	XP_021750095.1	208
CqGLP-9	AUR62028952	XP_021714979.1	439**
CqGLP-10*	AUR62025242	XP_021750069.1	193
CqGLP-12	AUR62028948	XP_021714976.1	208
CqGLP-13	AUR62025219	XP_021750074.1	208
CqGLP-14	AUR62025218	XP_021750075.1	208
CqGLP-15	AUR62025225	XP_021750080.1	208
CqGLP-16	AUR62025234	XP_021750050.1	208
CqGLP-17	AUR62025235	XP_021750126.1	208
CqGLP-18	AUR62025236	XP_021750060.1	208
CqGLP-19	AUR62028958	XP_021714981.1	208
CqGLP-20	AUR62040806	XP_021754444.1	208
CqGLP-21	AUR62028956	XP_021714980.1	208
Average size (aa) of quinoa GLPs			207

* Genes selected for qRT-PCR analysis

** Discarded from the average measurement due to the presence of putative introns, which might reveal an annotation error
